# Study protocol for a non-randomized trial of community participation support for people with Acquired Brain Injury who have ceased driving in Japan

**DOI:** 10.1371/journal.pone.0362487

**Published:** 2026-11-10

**Authors:** Satonori Nasu, Yu Ishibashi, Tsutomu Sasaki, Ryuji Kobayashi

**Affiliations:** 1 Sawarabi Facility, Department of Social Welfare, Nakaizu Rehabilitation Center, JA Kyosai, Izu City, Japan; 2 Department of Occupational Therapy, Graduate School of Human Health Sciences, Tokyo Metropolitan University, Tokyo, Japan; 3 Department of Rehabilitation, School of Health Sciences, Hokkaido Chitose College of Rehabilitation, Hokkaido, Japan; 4 Department of Occupational Therapy, School of Rehabilitation, Hyogo Medical University, Hyogo, Japan; Lady Hardinge Medical College, INDIA

## Abstract

**Introduction:**

Driving cessation after acquired brain injury (ABI) represents a major life transition that can restrict community participation and negatively affect quality of life. Although many individuals are unable to resume driving after illness or injury, structured support programs addressing life after driving cessation remain limited, and prospective evaluations are scarce. This study protocol describes a theoretically grounded community life support program for individuals with ABI who have ceased driving.

**Methods:**

This is a single-center, non-randomized controlled trial with ward-level cluster allocation conducted in a convalescent rehabilitation hospital in Japan. Participants with ABI who were determined to be unable to resume driving will be allocated at the ward level to either an intervention group receiving the Community Life Support Program for Individuals with Acquired Brain Injury after Driving Cessation (CLASP-AID) or a control group receiving usual care. CLASP-AID is a participant-centered program based on a person–occupation–environment framework and includes structured educational sessions for individuals and, when applicable, family members, information on alternative transportation, and training with alternative mobility devices. The primary outcome is occupational performance assessed using the Canadian Occupational Performance Measure, with the primary endpoint defined as the post-intervention COPM performance score. Secondary outcomes include readiness for mobility transition, health-related quality of life, depressive symptoms, environmental factors, and life-space mobility. The COPM, ARMT-J, SF-12, GDS-15, CEQ, and LSA will be assessed at baseline and post-intervention. When feasible, follow-up assessments will also be conducted at 1, 3, and 6 months after discharge to explore changes over time. LSA findings will be treated as exploratory because the baseline and post-intervention assessments are conducted during hospitalization and may be influenced by restrictions in life-space mobility associated with the inpatient environment.

**Discussion:**

This study will provide insight into the potential associations of a structured, occupation-focused support program for individuals with ABI after driving cessation. The findings are expected to inform future intervention development and contribute to evidence-based strategies for supporting community participation following driving cessation.

## 1 Introduction

Japan has experienced rapid population aging, with the proportion of older adults reaching a record high of approximately 29% [1]. Against this demographic background, stroke remains an important health concern in Japan, and cerebrovascular diseases, including stroke, are among the leading causes of death nationwide [2].

Stroke is known to cause a wide range of impairments, including physical, cognitive, sensory, and psychological impairments [3], which can result in substantial difficulties in daily life. Against this background, rehabilitation medicine has increasingly focused on automobile driving support for people after stroke.

A domestic survey of 525 stroke survivors reported that approximately 70% wished to resume driving at the time of hospital discharge [4]; A multicenter questionnaire survey of individuals with brain injury in Japan reported that approximately 58% had resumed driving, indicating that approximately 42% had not resumed driving at the time of the survey [5]. Previous studies have suggested that inability to resume driving after acquired brain injury is associated with reduced community participation and restricted life-space mobility [6], as well as depressive tendencies [7].

Furthermore, qualitative research indicates that for some individuals, the inability to resume driving is perceived not merely as a restriction on mobility but as a loss of independence and future life roles [8–10]. Such perceptions may influence psychological reactions following driving cessation, as well as how individuals receive and engage with support.

Recognizing these responses is clinically important, as support following driving cessation may need to address not only practical mobility challenges but also emotional and cognitive reactions that shape how individuals envision their future lives. At the same time, supporting adaptation after driving cessation does not necessarily require abandoning aspirations for future driving resumption. Rather, maintaining meaningful daily activities and social participation may help preserve motivation, health, and a sense of possibility during this transitional period.

A pilot study of individuals who had ceased driving after acquired brain injury found reductions in participation in desired and necessary activities among those whose driving cessation had been decided by healthcare professionals or family members [11]. Together, these findings indicate that experiences following driving cessation, particularly continued engagement in meaningful activities regardless of subsequent driving outcomes, may be important considerations when designing support strategies for individuals with acquired brain injury.

To inform the design of a driving cessation support program, we integrated findings from qualitative studies of individuals with acquired brain injury who had ceased driving and adopted the Person–Environment–Occupation model [12], a widely used framework in occupational therapy, to construct a middle-range theoretical framework [13].

In this framework, the “person” includes not only the individual who has ceased driving but also family members and close relatives who provide support. After driving cessation, community mobility often becomes difficult, and individuals frequently rely on family members for transportation. However, repeated requests for assistance may impose a burden on both the individual and family members, highlighting the importance of considering family involvement in post–driving cessation support [14,15].

The “occupation” component focuses on the meaning, role, and value that driving held for the individual. Identifying activities that have become difficult due to driving cessation and exploring alternative ways to continue these activities are considered essential [16].

The “environment” includes available public resources, alternative transportation options, services, and social supports. By individually examining which environmental resources can be utilized, support for individuals who have ceased driving may be more appropriately tailored [17].

To address this aim, we developed a participant-centered community life support program, the Community Life Support Program for Individuals with Acquired Brain Injury after Driving Cessation (CLASP-AID), based on a person–occupation–environment framework.

We hypothesize that participation in the CLASP-AID program will be associated with greater readiness for driving cessation and higher levels of performance in necessary post-discharge activities compared with usual care, which was selected as the comparator to evaluate the added value of a structured, occupation-centered program beyond existing rehabilitation support.

## 2 Participant selection

### 2.1 Target population

Participants will be individuals with acquired brain injury who are admitted to or attending outpatient services at a convalescent rehabilitation center in Shizuoka Prefecture, Japan, and who have undergone a driving assessment and been determined unable to resume automobile driving. The target population includes individuals aged 55 years or older, based on the age criteria used in previous studies of the Japanese version of the Assessment of Readiness for Mobility Transition (ARMT-J) [18].

### 2.2 Inclusion criteria

Participants must meet all of the following criteria:

1) Be aged 55 years or older.
2) Provide written informed consent after receiving sufficient explanation of the study and demonstrate sufficient cognitive ability to understand the study procedures and complete the study questionnaires and interviews
3) Have ceased driving with a standard automobile driver’s license.
4) Be independent in basic activities of daily living at the time of enrollment.

### 2.3 Exclusion criteria

Participants will be excluded if any of the following apply:

1) Presence of conditions defined by the Public Safety Commission as disqualifying for driving resumption, including epilepsy, schizophrenia, or dementia.
2) Evident cognitive impairment, defined as a Mini-Mental State Examination (MMSE) score of 24 or lower [19].
3) Aphasia that interferes with adequate understanding of study procedures and assessments.

### 2.4 Recruitment

Potential participants will be identified among inpatients and outpatients undergoing driving assessment at the study institution. Eligible individuals will be identified by the occupational therapist in charge of driving assessment, and invited to participate promptly after the attending physician and occupational therapist determine that driving cannot currently be safely resumed because of illness-related impairments. This determination follows the clinical driving assessment process and, in most cases, is made after the practical driving assessment. Written informed consent will be obtained before enrolment, baseline research assessment, and delivery of the study intervention, as described in Section 4.

To enhance questionnaire response rates, reminder contacts will be made by research staff when questionnaires are not returned within the expected timeframe. As of August 16, 2026, 27 participants had been enrolled, including 10 participants in the control group and 17 participants in the intervention group. Participant recruitment began on November 16, 2024, and was completed on September 30, 2026. The final follow-up is scheduled to be completed by March 31, 2027.

Clinical driving-assessment records will be reviewed to identify all individuals who underwent driving assessment during the recruitment period. Among those judged unable to resume driving at that time, study eligibility and subsequent enrolment will be ascertained using the criteria in Sects 2.2 and 2.3. Individuals who did not undergo driving assessment are outside this record-based screening population. The results report will include a participant flow diagram showing the numbers assessed for eligibility, eligible, consented and enrolled, and reasons for non-enrolment where documented. Post-enrolment exclusions, withdrawals, assessment completion, and loss to follow-up will be reported by group, with reasons where documented. Questionnaire return or nonreturn will be recorded at each scheduled follow-up. Nonresponse will be distinguished from withdrawal of consent, as described in Sect 5.5. Information that cannot be established from the available records will be identified as unavailable.

## 3 Sample size, data types and study settings

### 3.1 Sample size

Because this is a pilot and exploratory study conducted at a single rehabilitation center with allocation determined by ward admission, the sample size was determined primarily on the basis of recruitment feasibility. A formal power calculation for the planned ANCOVA was not performed because reliable prior estimates of the intervention-specific between-group effect and the correlation between baseline and post-intervention COPM performance scores were not available.

Based on the anticipated number of eligible patients at the study site, a target sample size of 30 participants with evaluable primary outcome data was planned. To account for incomplete assessments and loss to follow-up, up to 40 participants may be recruited. No fixed equal allocation between groups was assumed because group assignment is determined by ward admission.

This sample size is not intended to provide definitive statistical power for establishing intervention effectiveness or for accounting statistically for ward-level clustering. The primary analysis will instead estimate the adjusted between-group difference in post-intervention COPM performance scores and its 95% confidence interval. The resulting estimates of effect magnitude, variability, recruitment, retention, and data completeness will be used to inform the design and formal sample size calculation of a future adequately powered trial.

### 3.2 Types of data and materials

All data collected during the study will initially be recorded on paper-based case report forms and subsequently entered into a password-protected computer within the study institution for analysis.

### 3.3 Study setting

The CLASP-AID program and related assessments will be conducted in the occupational therapy department of the JA Kyosai Nakaizu Rehabilitation Center. If alternative mobility devices are required as substitutes for automobile driving, evaluations and interventions will be performed using the institution’s on-site mobility training course. Final evaluation of alternative mobility device use will be conducted with support from specialized external providers. When applicable, the Wheelchair Competency Test (WCT) will be used as a standardized assessment to support the evaluation process.

## 4 Informed consent

The study explanation and consent process will begin promptly after the attending physician and occupational therapist determine that driving cannot currently be safely resumed because of illness-related impairments. This determination is based on routine clinical driving assessments, including desk-based, driving simulator, and practical driving assessments, and is made after the practical driving assessment in most cases. It concerns the participant’s current ability to resume driving, rather than necessarily indicating permanent driving cessation. Written informed consent will be obtained from all participants before enrolment, baseline research assessment, and delivery of the study intervention. An occupational therapist will review the ethics committee-approved participant information and consent documents (S4 and S5 Files) together with the individual, provide oral explanations, and confirm the individual’s willingness to participate. These explanations include the research use of routine clinical assessment results, including the inpatient COPM. Eligibility requires sufficient cognitive ability to understand the study procedures and complete the assessments, as specified in Sections 2.2 and 2.3. Participants will be informed that participation is voluntary and that refusal or withdrawal will not result in any disadvantage. If new information arises that may affect a participant’s decision to continue in the study, or if the study protocol is modified, participants will be promptly informed. Revised consent documents will be approved by the ethics committee, and re-consent will be obtained as necessary. Oral explanations will be documented in the medical record, including the date, method of explanation, personnel involved, and the participant’s decision.

## 5 Materials and methods

### 5.1 Ethics approval

The original ethics application was submitted to the Institutional Ethics Committee of Nakaizu Rehabilitation Center on August 6, 2024, and approved on October 1, 2024. Although the planned study period was scheduled to begin on September 1, 2024, no participants were enrolled and no study-related data were collected before ethics approval. The first participant was enrolled on November 16, 2024. The current protocol (version 1.0, dated January 16, 2026) was subsequently approved by the same committee (Approval No. 社中総発第77号). Written informed consent was obtained from all participants before enrollment.

### 5.2 Trial registration

This trial was retrospectively registered with the UMIN Clinical Trials Registry on March 25, 2025 (UMIN000057350). The first participant was enrolled on November 16, 2024. The research team had initiated the registration process before participant recruitment and believed that all required registration procedures had been completed. However, it was subsequently identified that the registration had not been finalized and publicly disclosed. Once this was recognized, the remaining procedures were completed promptly. Participant recruitment began on November 16, 2024, and was completed on September 30, 2026. The final follow-up is scheduled to be completed by March 31, 2027. The authors confirm that all ongoing and related trials for this intervention are registered.

### 5.3 Study design and intervention

#### 5.3.1 Study design.

This study is a single-center, non-randomized controlled trial with a cluster allocation design. Participants will be assigned to either an intervention group (Group A) or a control group (Group B) based on ward-level allocation. The intervention group will receive the Community Life Support Program for Individuals with Acquired Brain Injury after Driving Cessation (CLASP-AID), while the control group will receive usual care.

Because this study is conducted in a post-acute inpatient rehabilitation hospital in Japan, complete randomization and blinding are not feasible. To minimize contamination between groups, allocation will be performed at the ward level, with one ward assigned to the intervention group and another to the control group. This study involves no invasive procedures.

This exploratory study will estimate between-group differences in outcomes. Because allocation is non-randomized and determined by ward admission, these differences may reflect both the intervention and differences in patient characteristics or ward-level care. Baseline characteristics will be summarized by group to describe potential imbalances arising from the non-randomized allocation.

#### 5.3.2 Allocation.

Participants admitted to the second-floor ward will be assigned to the control group, whereas those admitted to the third-floor ward will be assigned to the intervention group. Admission to the second- or third-floor ward is determined by a nurse-led team responsible for ward management, based on bed availability and the balance of patients’ clinical conditions across the wards. The investigators do not determine or alter ward placement for study group allocation. Ward admission is not randomized and cannot be assumed to be independent of patient characteristics.

Participants are informed that the study compares two different types of support and that they will receive the support routinely provided in their assigned ward. They are not explicitly informed whether their ward is designated as the intervention or control group. However, because of the nature of the educational intervention, participant blinding cannot be ensured.

#### 5.3.3 Intervention program: CLASP-AID.

The Community Life Support Program for Individuals with Acquired Brain Injury after Driving Cessation (CLASP-AID) consists of four educational sessions for individuals with acquired brain injury and, when applicable, one educational session for family members. The first individual session, which addresses driving support provided by the institution, is provided to both the intervention and control groups as part of usual care. The four individual sessions are delivered face-to-face on a one-to-one basis, with each session lasting approximately 40–60 minutes. The content of the individual and family sessions is organized into five modules, as summarized in Table 1.

**Table 1:** Modules and content of the CLASP-AID program.

Module	Module title	Key contents
1	Understanding driving and mobility support	(1) Overview of driving assessment and support pathways within the rehabilitation center(2) Legal and regulatory systems related to post-injury driving in Japan(3) Physical impairments and their potential impact on driving(4) Cognitive impairments and their potential impact on driving(5) Role and purpose of driving simulators in assessment and training(6) On-road driving assessment procedures(7) Re-evaluation processes when driving resumption is not immediately feasible
2	Adapting to driving cessation	(1) Role of the rehabilitation center in supporting individuals after driving cessation(2) Identification of individual challenges associated with driving cessation, including symptom-related difficulties(3) Promotion of stable and meaningful daily routines to support health, frailty prevention, and community participation(4) Strategies for daily life management following driving cessation
3	Exploring alternative mobility options	(1) Public transportation options (e.g., bus, taxi, train)(2) Bicycles and adapted cycles (e.g., tricycles, four-wheeled cycles)(3) Mobility scooters(4) Powered wheelchairs(5) Transportation support provided by family members or others(6) Community-based transportation and support services
4	Reconstructing daily life after driving cessation	(1) Identifying activities that have become challenging following illness or injury(2) Clarifying difficulties arising from driving cessation in daily life(3) Developing practical strategies to address identified challenges(4) Setting short-term goals and action plans prior to re-evaluation
5	Family education and support	(1) Potential challenges experienced by individuals following driving cessation, including health-related changes(2) Potential challenges experienced by family members, such as transportation burden and schedule management(3) Approaches to supporting and communicating with individuals who have ceased driving

Although the core modules are common to all participants in the intervention group, the specific information and support provided are tailored to each participant’s identified occupational needs, living environment, available transportation resources, and preferences.

The family session focuses on key points for support by family members and close relatives. The program additionally includes the organization and provision of information on local services and public transportation, including pamphlets on available mobility resources in the surrounding area. Assessment and training in the use of alternative mobility devices, such as senior scooters and powered wheelchairs, are also included as part of the intervention. The CLASP-AID educational materials are currently available in Japanese and may be obtained from the corresponding author upon reasonable request.

No restrictions are placed on concomitant rehabilitation or community services during the trial. For concomitant services, the study will record long-term care insurance status and post-discharge service use at the post-intervention assessment and at 1, 3, and 6 months after discharge.

During the intervention period, CLASP-AID educational materials will be provided only to participants in the intervention group. The therapist responsible for each participant will document the support provided in the participant’s clinical record. No dedicated intervention fidelity checklist is included in the study procedures.

#### 5.3.4 Intervention period.

The schedule of enrolment, interventions, and outcome assessments is summarized in Fig 1. The study period extends from September 1, 2024, to March 31, 2027. The study period was extended because participant recruitment was slower than anticipated. This extension was approved by the Institutional Ethics Committee of Nakaizu Rehabilitation Center on January 14, 2026 (Approval No. 社中総発第55号).

**Fig 1:**
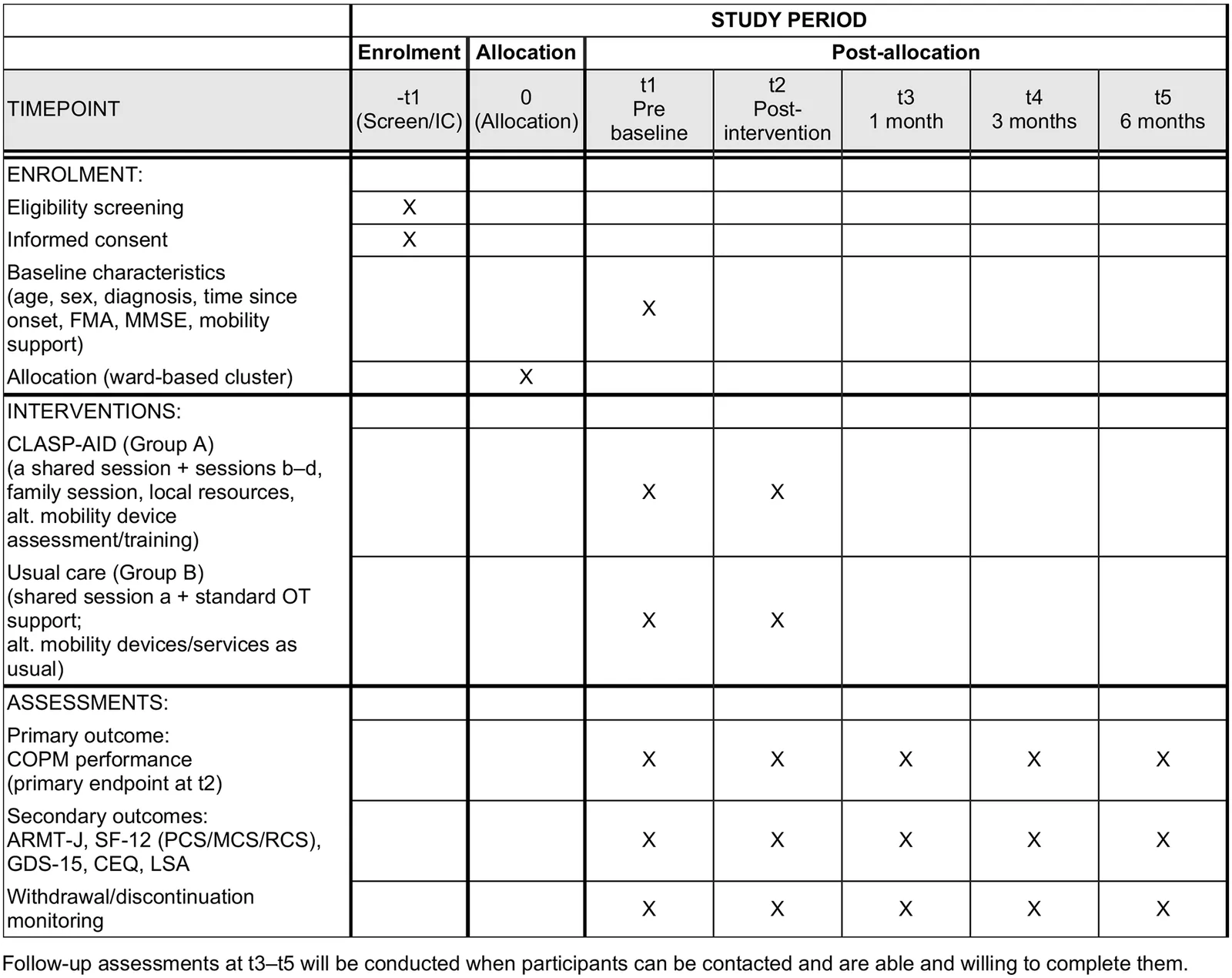
Schedule of enrolment, interventions, and assessments (SPIRIT figure).

#### 5.3.5 Timing of the intervention.

Following completion of the driving assessment and a multidisciplinary conference involving the physician and occupational therapist, the intervention components specific to the intervention group are initiated only after written informed consent and the baseline research assessment have been completed. After this conference, the program is explained to the participant and, when applicable, to family members, and the intervention is implemented.

The first educational session (session a), which is conducted at the start of the driving assessment as part of usual care, is provided to both the intervention and control groups. Subsequently, participants in the intervention group receive sessions (b)–(d) over approximately one week. A family session (session e) is provided when applicable, depending on the availability and involvement of family members.

#### 5.3.6 Intervention content and group differences.

Participants in the intervention group receive the full CLASP-AID program, whereas participants in the control group receive usual care delivered by their assigned occupational therapist. Usual care is provided within routine occupational therapy and is individualized according to each participant’s clinical needs and the therapist’s judgment; its content, frequency, and duration are not standardized. The structured CLASP-AID educational materials are not used in the control group, and details of the intervention program are not routinely shared with occupational therapists providing usual care. In both groups, alternative mobility devices and mobility support services may be provided in accordance with usual clinical practice. If requested, the CLASP-AID educational materials will be made available to control-group participants after completion of the study. This option is explained in the informed consent document, which instructs participants who wish to receive the materials to contact their treating therapist or the principal investigator. The document provides the principal investigator’s name, email address, and telephone number.

#### 5.3.7 Intervention providers.

The CLASP-AID intervention is delivered by either the participant’s assigned occupational therapist or an occupational therapist involved in driving assessment and support at the study institution. All participants in the intervention group receive the four planned individual sessions, and the family session is provided when applicable. Before delivering the intervention, participating occupational therapists receive training on the outcome assessment procedures and on how to use the CLASP-AID educational materials to support participants after driving cessation.

### 5.4 Outcome assessment procedures

#### 5.4.1 Assessment schedule and administration.

The date on which the attending physician and occupational therapist determine, during a conference, that driving cannot currently be safely resumed because of illness-related impairments serves as the reference date for the baseline assessment. The baseline (pre-intervention) assessment is conducted after written informed consent, promptly following this determination, generally within one week of the reference date. The COPM, ARMT-J, SF-12, GDS-S-J (GDS-15), CEQ, and LSA will be administered at baseline and post-intervention during hospitalization. After discharge, these measures will be collected using mailed questionnaires at 1, 3, and 6 months for participants who can be contacted and are able and willing to complete them. The assessment tools and the adapted postal follow-up procedure for the COPM are described below.

Where feasible, the roles of intervention providers, outcome assessors, and data analysts are separated. However, depending on staff availability, inpatient COPM assessments are conducted either by the occupational therapist who delivers the intervention or by another occupational therapist. Therefore, COPM assessors are not consistently independent of the intervention and are not blinded to group allocation.

#### 5.4.2 Canadian occupational performance measure.

The Canadian Occupational Performance Measure (COPM) is a semi-structured interview designed to identify and evaluate self-perceived problems in occupational performance [20]. During hospitalization, the COPM will be administered through an interview as part of routine clinical care. In the present study, the COPM is applied within the context of driving cessation to capture occupational performance problems associated with the loss of driving. During the interview, participants who have discontinued driving are asked to identify activities and roles that they want or need to perform but experience difficulty with as a consequence of driving cessation, including activities of daily living, community mobility, and social participation. The interview covers occupational performance problems associated with driving cessation using the COPM rating scales.

Participants select up to five occupational performance problems that they consider most important and rate their perceived performance and satisfaction for each item using the standard 10-point ordinal scale, with higher scores indicating better perceived performance and greater satisfaction [21]. The mean performance and satisfaction scores across the selected items will be calculated for each participant.

At both inpatient and post-discharge COPM reassessments, participants are reminded of the previously identified activities so that they can re-rate those activities. For postal follow-up, the form will include these activity descriptions and a designated space for participants to record their current performance and satisfaction ratings. These follow-up ratings will be self-completed by participants rather than obtained through a therapist-administered interview. Previous COPM scores, in contrast to activity descriptions, are not routinely shown or otherwise disclosed at either stage; they may be disclosed only if a participant explicitly asks for them. The postal format is an adapted follow-up procedure, and the measurement properties of the standard interview-based COPM cannot be assumed to apply unchanged. Follow-up COPM findings will be interpreted as exploratory.

#### 5.4.3 Readiness for mobility transition.

Readiness for mobility transition will be assessed using the Japanese version of the Assessment of Readiness for Mobility Transition (ARMT-J) [18]. The ARMT is a self-report questionnaire designed to evaluate individuals’ emotional and attitudinal readiness when changes in mobility are required due to aging or other factors [22]. The ARMT-J consists of 11 items across three subscales: Anticipatory Anxiety (AA), Perceived Burden (PB), and Adverse Situations (AS). Each item is rated on a 5-point Likert scale ranging from 1 (“strongly disagree”) to 5 (“strongly agree”), and scores are interpreted using the mean score of each subscale.

#### 5.4.4 Health related quality of life.

Health-related quality of life will be assessed using the Medical Outcomes Study 12-Item Short-Form Health Survey, version 2 (SF-12) [23]. The SF-12 is a reliable and valid instrument that yields eight subscale scores: physical functioning, role physical, bodily pain, general health, vitality, social functioning, role emotional, and mental health. These subscales are summarized into three component scores: the Physical Component Summary (PCS), Mental Component Summary (MCS), and Role/Social Component Summary (RCS) [24]. Higher scores indicate better health-related quality of life.

#### 5.4.5 Depressive symptoms.

Depressive symptoms will be assessed using the Geriatric Depression Scale–Short Version–Japanese (GDS-S-J; GDS-15) [25], which was derived from the short version of the Geriatric Depression Scale developed for screening depressive symptoms in older adults [26,27]. The GDS-S-J consists of 15 items selected from the original 30-item GDS, with dichotomous (“yes”/“no”) response options. Total scores range from 0 to 15, with higher scores indicating more severe depressive symptoms.

#### 5.4.6 Environmental factors.

Environmental factors will be assessed using the Comprehensive Environmental Questionnaire for the Elderly (CEQ) [28,29]. The CEQ is a validated questionnaire consisting of 14 items across three domains: safe living environment (6 items), social interaction environment (6 items), and family environment (2 items). Each item is rated on a 4-point scale ranging from 1 (“not at all”) to 4 (“sufficient”). Domain scores and a total score are calculated by simple summation, with higher scores indicating greater satisfaction with the environment.

#### 5.4.7 Life space mobility.

Life-space mobility will be assessed using the Life-Space Assessment (LSA) [30,31]. The LSA evaluates mobility by quantifying the spatial extent of an individual’s movement, ranging from within the home to locations beyond the local community, while incorporating information on movement frequency and level of independence. Participants report their mobility over the previous four weeks, including whether they reached each level of life-space, how often they did so, and whether assistive devices or personal assistance were required. Total scores range from 0 to 120, with higher scores indicating greater life-space mobility. The original LSA has evidence of reliability and validity, and a Japanese study supports the validity of its composite scores [30,31].

Because the baseline and post-intervention assessments are conducted during hospitalization, the corresponding LSA scores may be influenced by restrictions in life-space mobility associated with the inpatient environment. Therefore, LSA findings will be treated as exploratory and interpreted cautiously.

#### 5.4.8 Evaluation of electric mobility devices.

Practice and evaluation of electric mobility devices will be conducted using the mobility course available at the study institution. If participants plan to use an electric mobility device in the community, final evaluation and judgment regarding device use will be conducted with support from specialized external providers.

#### 5.4.9 Primary outcome.

The primary outcome is the mean COPM performance score across the participant-selected occupational performance problems. The primary endpoint is the post-intervention COPM performance score, with the primary between-group comparison adjusted for the baseline score as described in the statistical analysis plan. COPM follow-up scores at 1, 3, and 6 months after discharge will be used in exploratory longitudinal analyses.

#### 5.4.10 Secondary outcomes.

Secondary outcomes are readiness for mobility transition (ARMT-J subscale scores), health-related quality of life (SF-12 scores), depressive symptoms (GDS-S-J total score), environmental factors (CEQ domain and total scores), life-space mobility (LSA total score), and evaluation of electric mobility devices. The assessment procedures and time points are described in Sect 5.4. In this study, the secondary patient-reported and functional outcomes are included as exploratory assessments to evaluate feasibility and inform future studies, rather than as confirmatory measures of intervention effectiveness. In particular, LSA findings will be interpreted cautiously because the inpatient environment may restrict life-space mobility at baseline and post-intervention.

#### 5.4.11 Baseline characteristics.

The following baseline characteristics will be collected for descriptive purposes:

(1) Age
(2) Sex
(3) Diagnosis
(4) Time since onset
(5) Availability of mobility support from family members or caregivers
(6) Upper- and lower-extremity motor function assessed using the Fugl-Meyer Assessment (FMA) motor subscales [32]. Upper-extremity (FMA-UE; range, 0–66) and lower-extremity (FMA-LE; range, 0–34) scores will be recorded separately, with higher scores indicating better motor function. Pre-intervention scores and dates will be extracted from clinical records. Unperformed assessments will be recorded as missing, with reasons where available, not assigned maximum scores solely because paresis is absent. FMA requires an examiner and is not included in the 1-, 3-, or 6-month postal follow-ups.
(7) Cognitive function (Mini-Mental State Examination; MMSE) [19]

### 5.5 Study discontinuation and withdrawal

**Discontinuation criteria**

Participants will be withdrawn from the study if any of the following conditions apply:

- The participant requests to withdraw from the study or withdraws informed consent.
- The participant is found, after enrollment, not to meet the inclusion criteria.
- Worsening of the primary disease makes continuation of outcome assessment inappropriate, as judged by the attending physician.
- Exacerbation of comorbid conditions makes continuation of outcome assessment difficult

**Dropout criteria**

Participants will be considered dropouts if they die during the study period.

When feasible, participants who discontinue the intervention will be invited to complete outcome assessments scheduled at the time of discontinuation.

Failure to return questionnaires at some or all of the three scheduled post-discharge follow-up assessments does not, by itself, constitute withdrawal of consent or study discontinuation. Unless a participant requests withdrawal or meets another discontinuation or dropout criterion listed above, questionnaires will continue to be mailed at the remaining scheduled time points. A missed assessment does not preclude participation at a later follow-up. Unreturned questionnaires will be recorded as missing at the corresponding time points. Participants will not be excluded from analysis solely because of follow-up nonresponse; available data will be used in accordance with the statistical analysis plan in Sect 5.8.

### 5.6 Safety monitoring and adverse events

Potential adverse events include fatigue or psychological distress during the educational sessions and falls, collisions, or injuries during powered wheelchair or senior mobility scooter assessment and training. Participants are monitored by the occupational therapist throughout each session. If fatigue, distress, or physical discomfort occurs, the session will be paused, rest will be provided, and discontinuation of the intervention or study participation will be considered when necessary.

Powered mobility assessments are conducted only on the institution’s designated mobility course or driving course when no other vehicles are operating. An occupational therapist remains beside the participant and provides immediate assistance when necessary. The therapist controls speed before steps and slopes to reduce the risk of falls, and a helmet is made available to participants who wish to use one.

If an accident occurs outdoors, staff will immediately contact the rehabilitation center by mobile phone and request additional assistance. The event will subsequently be managed and reported in accordance with the institutional incident-reporting procedures of the Safety Management Committee. Compensation for injuries will be handled through the institution’s insurance coverage, when applicable.

The principal investigator monitors participant recruitment, completion of outcome assessments, protocol deviations, and adverse events throughout the study. Any safety incidents are managed and reported in accordance with the institutional procedures of the Safety Management Committee. An independent data monitoring committee was not established because this is a small, single-center study involving a non-invasive educational and occupational therapy intervention with minimal anticipated risk.

At each educational or powered mobility session, the occupational therapist monitors participants for adverse events through direct observation and participant self-report. Any observed or spontaneously reported adverse event is documented in the participant’s clinical record. Accidents and other events requiring institutional reporting are additionally documented through the institutional incident-reporting system. The principal investigator assesses the severity of each event and its possible relationship to the study intervention. Serious or unexpected events are reported to the Institutional Ethics Committee and the Safety Management Committee in accordance with institutional procedures.

### 5.7 Burden, risks, and benefits

#### 5.7.1 Anticipated benefits.

This intervention study is not expected to provide direct benefits to participants, as evidence of intervention effectiveness is not yet established. However, the findings may contribute to future improvements in rehabilitation and community mobility support. Participants who complete all three post-discharge questionnaires (1, 3, and 6 months) will receive a single 1,000-JPY QUO gift card after the final assessment.

#### 5.7.2 Anticipated burdens and risks.

Participants in the intervention group will receive an additional educational program focused on driving cessation support. Although participation may involve some burden, similar follow-up support has previously been provided as part of routine clinical practice. If participants experience fatigue or discomfort during the program, appropriate rest will be provided, and study discontinuation will be considered as needed. No serious adverse events related to the intervention are anticipated, as the CLASP-AID program is a non-invasive, educational intervention. Any adverse events or unintended effects reported spontaneously by participants or observed by clinical staff during the study period will be documented by the attending physician or occupational therapist. Serious adverse events, if any occur, will be promptly reported to the institutional ethics committee in accordance with institutional policies.

### 5.8 Statistical analysis

All statistical analyses will be performed using R version 4.5.2 (R Foundation for Statistical Computing, Vienna, Austria). The distributions of continuous variables will be assessed using histograms, quantile–quantile plots, and the Shapiro–Wilk test. Approximately normally distributed variables will be summarized using means and standard deviations, whereas markedly skewed variables will be summarized using medians and interquartile ranges. Categorical variables will be summarized using frequencies and percentages.

Given the pilot and exploratory nature of this study, the analyses are intended to estimate the magnitude and precision of potential between-group differences rather than to provide confirmatory evidence of intervention effectiveness. Effect estimates will therefore be presented with 95% confidence intervals, and statistical tests will be interpreted as exploratory.

Primary analyses will focus on the post-intervention COPM performance outcome. The adjusted between-group difference will be estimated using analysis of covariance (ANCOVA), with the post-intervention COPM performance score as the dependent variable, group as the main explanatory variable, and baseline COPM performance score as a covariate. The adjusted mean difference and its 95% confidence interval will be reported.

Baseline characteristics, including age, sex, diagnosis, time since onset, MMSE score, and availability of mobility support from family members or caregivers, will be summarized by group to describe potential imbalances arising from the non-randomized allocation. As a sensitivity analysis, a parsimonious ANCOVA model additionally adjusted for age and MMSE score will be examined. These covariates were selected because of their clinical relevance to occupational performance and their potential association with the study outcome. Propensity score matching will not be performed because the small sample size may result in unstable propensity score estimation, inadequate overlap, and further loss of participants through matching. Moreover, because intervention allocation corresponds directly to ward allocation and only two wards are included, propensity score methods cannot separate the intervention effect from unmeasured ward-level effects. The adjusted estimates will therefore be interpreted with these potential sources of confounding in mind.

The assumptions of the ANCOVA model will be evaluated using residual and quantile–quantile plots. If substantial non-normality, influential outliers, or heteroscedasticity is identified, a rank-based ANCOVA will be conducted as a sensitivity analysis. For exploratory comparisons of secondary outcomes with markedly non-normal distributions, nonparametric methods, such as the Mann–Whitney U test for between-group comparisons and the Wilcoxon signed-rank test for within-group comparisons, will be considered as appropriate. Results of these analyses will be interpreted as exploratory.

As an exploratory analysis, changes over time from baseline to post-intervention and the available 1-, 3-, and 6-month follow-up assessments will be evaluated using longitudinal COPM performance data. The model will include fixed effects for group, time, and the group-by-time interaction, with a participant-specific random intercept to account for repeated measurements within individuals. Because only two wards are included, ward-level clustering cannot be modeled reliably, and the longitudinal analyses will be interpreted as exploratory.

Secondary outcomes will be summarized by group and assessment time point using appropriate descriptive statistics. Where applicable, between-group estimates and their 95% confidence intervals will be presented. Because the secondary outcomes are exploratory, no confirmatory conclusions will be based on these analyses. If exploratory hypothesis tests are conducted across multiple secondary outcomes, p-values will be adjusted using the Holm method within each assessment time point, and both unadjusted and adjusted p-values will be reported. Exploratory analyses of associations between ARMT-J scores and baseline characteristics will be reported separately, with Holm adjustment applied across the tested associations.

The extent and reasons for missing data will be summarized by group and assessment time point. The primary analysis will follow a modified intention-to-treat principle, including participants according to their ward-based group assignment regardless of adherence to the intervention. For participants with a baseline COPM assessment but a missing post-intervention COPM assessment, missing primary outcome values will be addressed using multiple imputation under a missing-at-random assumption. The imputation model will include group, baseline COPM performance score, age, MMSE score, and available post-baseline COPM measurements. A complete-case ANCOVA will be conducted as a sensitivity analysis.

For the longitudinal mixed-effects model, all available COPM measurements will be used, and missing follow-up observations will be handled through maximum-likelihood estimation under a missing-at-random assumption. Exploratory secondary outcomes will be analyzed using available observations without imputation, and the number of observations contributing to each analysis will be reported. Last observation carried forward will not be used.

## 6 Data management and confidentiality

All data obtained in this study will be used exclusively for research purposes and handled in accordance with confidentiality requirements. Paper-based records will be securely stored in locked cabinets at the study institution under the supervision of the institutional ethics committee, and electronic data will be stored on password-protected computers. Data reported in academic conferences and journals will be anonymized and presented in aggregated form to prevent identification of individual participants. When data are shared with external collaborating researchers, all personally identifiable information will be encrypted to prevent data breaches. Participants may request disclosure of their personal information; in such cases, relevant study documents will be made available to the extent that disclosure does not compromise the privacy of other participants or the scientific integrity of the study.

### 6.1 Data management and quality control

Data collected on paper-based assessment forms are entered into an electronic spreadsheet database by one research staff member. A second staff member independently verifies the entered data against the original source documents, and any discrepancies identified during verification are resolved by referring to the source documents. The principal investigator oversees the data management and verification procedures.

The analysis dataset is pseudonymized using unique study identification numbers and does not contain direct personal identifiers. The participant identification list linking study identification numbers to personal information is stored separately from the research dataset. Electronic records are stored on password-protected media, and paper documents and storage media are kept in a locked cabinet.

Standardized data collection forms are used for all questionnaire-based assessments.

### 6.2 Future use of data

Data obtained in this study may be used for future research that cannot be specified at the time of consent, including larger-scale studies, provided that participants receive a new explanation of the proposed research and give renewed consent before such use. Any future research use will require approval from the institutional ethics committee. Participants may withdraw consent for future use at any time, and their data will be destroyed in accordance with their wishes.

### 6.3 Data storage and disposal

Unless participants have consented to long-term storage for future research, study data will be retained for at least five years after study completion or three years after publication of study results, whichever is later. Paper records will be destroyed by shredding, and electronic data will be permanently deleted and rendered unrecoverable.

## 7 Discussion

This study protocol describes a non-randomized controlled trial designed to examine associations between participation in a community life support program for individuals with acquired brain injury after driving cessation and changes in occupational performance and related outcomes. Controlled evaluations of interventions supporting driving retirement or cessation in older adults remain limited [33].

CarFreeMe TI is a community-based group education and support program addressing driving cessation and community mobility [34,35]. The present study examines an occupation-centered intervention delivered in a convalescent rehabilitation setting. Informed by a person–occupation–environment framework [13], the CLASP-AID program integrates individualized goal setting, family involvement, and context-specific mobility planning. By prospectively following participants across the transition from rehabilitation to community living, this study aims to provide insight into how occupation-focused support may be associated with changes in occupational performance　and readiness for mobility transition after discharge.

Importantly, the present approach does not frame driving resumption and driving cessation as mutually exclusive outcomes. Qualitative studies have described driving cessation as a highly disruptive life event, often accompanied by emotional distress, changes in identity, and a reduced sense of meaning in daily life [8–10]. In addition, driving cessation has been associated with restricted life-space mobility and reduced opportunities for social participation, which may contribute to increased vulnerability to frailty-related outcomes. From this perspective, supporting meaningful daily activities, social engagement, and alternative mobility options after driving cessation is considered essential regardless of driving status.

At the same time, this approach does not discourage aspirations for driving resumption. For individuals who continue to express a desire to resume driving, establishing a stable and fulfilling daily life may also provide a supportive foundation for maintaining motivation, health, and functional readiness. Thus, the CLASP-AID program aims to mitigate the negative consequences associated with driving cessation while preserving future mobility possibilities, rather than framing driving resumption as an all-or-nothing outcome.

Methodologically, this study adopts a non-randomized, cluster allocation design to minimize contamination within a single rehabilitation facility. Although the absence of randomization and blinding introduces important methodological limitations, ward-level allocation enhances feasibility and reflects real-world clinical conditions. Baseline adjustment is planned to partially address observed between-group differences; unmeasured patient characteristics and ward-level care may still contribute to the observed differences in outcomes.

Several limitations should be acknowledged. The pilot and exploratory nature of this study may limit statistical power, and the single-center design may constrain generalizability. Because ward admission takes account of bed availability and patients’ clinical conditions, selection bias and baseline differences between groups may arise. Because intervention assignment corresponds directly to ward allocation, intervention effects cannot be separated from unmeasured ward-level differences. Although baseline adjustment and sensitivity analyses are planned, residual confounding is likely, and observed between-group differences cannot be attributed to the program alone.

In some cases, the COPM assessor is the same occupational therapist who delivers the intervention. Because assessor independence and blinding are not consistently maintained, expectancy effects and assessor-related measurement bias cannot be excluded, particularly because COPM performance and satisfaction scores are based on participants’ self-ratings. Previous COPM scores may be disclosed upon a participant’s explicit request during either inpatient or post-discharge reassessment, although they are not routinely provided. Such disclosure may influence subsequent ratings and is a potential source of measurement bias in both the post-intervention and follow-up assessments. In addition, post-discharge COPM ratings are self-completed using mailed forms rather than obtained through therapist-administered interviews. This difference in administration mode may limit comparability across time points and will be considered when interpreting the exploratory longitudinal findings. Intervention delivery will be documented by the responsible therapists in routine clinical records without a dedicated intervention fidelity checklist. The level of detail in these records may vary between therapists, which may limit consistent verification of the delivery of individual intervention components. Because the baseline and post-intervention LSA assessments are conducted during hospitalization, scores at these time points may be influenced by restrictions in life-space mobility associated with the inpatient environment. In addition, because post-discharge follow-up assessments are conducted only for participants who can be contacted and are able and willing to complete them, the longitudinal findings may be affected by attrition and selection bias. These issues should be considered when interpreting changes in LSA scores over time. These limitations should be considered when interpreting the study findings. Nevertheless, the use of validated instruments and repeated assessments is expected to support the interpretability of the findings.

## Supporting information

S1 File SPIRIT checklist. (DOCX)

S2 File Ethics-approved protocol (original Japanese version). (DOCX)

S3 File Ethics-approved protocol (complete English translation). (DOCX)

S4 File Participant information sheet, consent form, and withdrawal form (original Japanese version). (DOCX)

S5 File Participant information sheet, consent form, and withdrawal form (English translation). (DOCX)
