# Supplementary material for: Study protocol for a non-randomized trial of community participation support for people with Acquired Brain Injury who have ceased driving in Japan
Source: PLoS One. 2026 Nov 10;21(11):e0362487. doi: 10.1371/journal.pone.0362487 (PMC13750569; doi:10.1371/journal.pone.0362487)
Supplement: Supplementary file 1 [file pone.0362487.s001.docx]

S1 SPIRIT Checklist

SPIRIT 2013 Checklist: Recommended items to address in a clinical trial protocol and related documents*

| Section/item | Item  No | Description | Addressed on  (page number) |
| --- | --- | --- | --- |
| **Administrative information** | | |  |
| Title | 1 | Descriptive title identifying the study design, population, interventions, and, if applicable, trial acronym | Title page (p. 1). |
| Trial registration | 2a | Trial identifier and registry name. If not yet registered, name of intended registry | Section 5.2, Trial Registration (pp. 14–15); registry identifier also on p. 1. |
|  | 2b | All items from the World Health Organization Trial Registration Data Set | UMIN-CTR registration record (UMIN000057350); Section 5.2, Trial Registration (pp. 14–15). |
| Protocol version | 3 | Date and version identifier | Section 5.1, Ethics Approval (p. 14): protocol version 1.0, dated January 16, 2026. |
| Funding | 4 | Sources and types of financial, material, and other support | Funding information is provided in the submission system in accordance with PLOS ONE requirements and is not included in the manuscript. |
| Roles and responsibilities | 5a | Names, affiliations, and roles of protocol contributors | Title page (p. 1); author contributions are provided in the submission system. |
|  | 5b | Name and contact information for the trial sponsor | Investigator-initiated study. Satonori Nasu, Nakaizu Rehabilitation Center, is responsible for study initiation and management. Contact details are provided in S2 File, Section 1-3, and S3 File, Section 1.3. |
|  | 5c | Role of study sponsor and funders, if any, in study design; collection, management, analysis, and interpretation of data; writing of the report; and the decision to submit the report for publication, including whether they will have ultimate authority over any of these activities | The role of the sponsor and funders is described in the Funding Information section of the submission system in accordance with PLOS ONE requirements. |
|  | 5d | Composition, roles, and responsibilities of the coordinating centre, steering committee, endpoint adjudication committee, data management team, and other individuals or groups overseeing the trial, if applicable (see Item 21a for data monitoring committee) | Sections 5.6 (pp. 31–33) and 6.1 (pp. 39–40): principal investigator oversight, safety monitoring, and data verification. Contributor roles are provided in the submission system. |
| Introduction |  |  |  |
| Background and rationale | 6a | Description of research question and justification for undertaking the trial, including summary of relevant studies (published and unpublished) examining benefits and harms for each intervention | Section 1, Introduction (pp. 4–7). |
|  | 6b | Explanation for choice of comparators | Section 1 (pp. 4–7) and Section 5.3.6 (pp. 21–22): rationale and usual-care comparator. |
| Objectives | 7 | Specific objectives or hypotheses | Section 1, final paragraph (p. 7). |
| Trial design | 8 | Description of trial design including type of trial (eg, parallel group, crossover, factorial, single group), allocation ratio, and framework (eg, superiority, equivalence, noninferiority, exploratory) | Sections 5.3.1–5.3.2 (pp. 15–16); Section 3.1 (pp. 10–11): non-randomized, exploratory ward allocation without a fixed equal allocation ratio. |
| Methods: Participants, interventions, and outcomes | | |  |
| Study setting | 9 | Description of study settings (eg, community clinic, academic hospital) and list of countries where data will be collected. Reference to where list of study sites can be obtained | Sections 2.1 (pp. 7–8), 3.3 (p. 12), and 5.3.1 (pp. 15–16). |
| Eligibility criteria | 10 | Inclusion and exclusion criteria for participants. If applicable, eligibility criteria for study centres and individuals who will perform the interventions (eg, surgeons, psychotherapists) | Sections 2.1–2.3 (pp. 7–8); intervention providers in Section 5.3.7 (p. 22). |
| Interventions | 11a | Interventions for each group with sufficient detail to allow replication, including how and when they will be administered | Sections 5.3.3–5.3.7 (pp. 17–22); Table 1 (pp. 18–19). |
|  | 11b | Criteria for discontinuing or modifying allocated interventions for a given trial participant (eg, drug dose change in response to harms, participant request, or improving/worsening disease) | Section 5.5 (pp. 30–31); safety-related interruption in Section 5.6 (pp. 31–33). |
|  | 11c | Strategies to improve adherence to intervention protocols, and any procedures for monitoring adherence (eg, drug tablet return, laboratory tests) | Sections 5.3.3 (pp. 19–20) and 5.3.7 (p. 22): tailored support, materials, therapist training, and clinical documentation; no dedicated intervention fidelity checklist is included. |
|  | 11d | Relevant concomitant care and interventions that are permitted or prohibited during the trial | Sections 5.3.3 (p. 20) and 5.3.6 (pp. 21–22): concomitant rehabilitation, community services, and usual care. |
| Outcomes | 12 | Primary, secondary, and other outcomes, including the specific measurement variable (eg, systolic blood pressure), analysis metric (eg, change from baseline, final value, time to event), method of aggregation (eg, median, proportion), and time point for each outcome. Explanation of the clinical relevance of chosen efficacy and harm outcomes is strongly recommended | Section 5.4 (pp. 23–30), including primary and secondary outcomes in Sections 5.4.9–5.4.10 (pp. 28–29); statistical analysis in Section 5.8 (pp. 34–38). |
| Participant timeline | 13 | Time schedule of enrolment, interventions (including any run-ins and washouts), assessments, and visits for participants. | Fig 1 (submitted separately); Sections 5.3.4–5.3.5 (pp. 20–21), 4 (pp. 12–13), and 5.4.1 (p. 23). |
| Sample size | 14 | Estimated number of participants needed to achieve study objectives and how it was determined, including clinical and statistical assumptions supporting any sample size calculations | Section 3.1 (pp. 10–11). |
| Recruitment | 15 | Strategies for achieving adequate participant enrolment to reach target sample size | Section 2.4 (pp. 9–10). |
| **Methods: Assignment of interventions (for controlled trials)** | | |  |
| Allocation: |  |  |  |
| Sequence generation | 16a | Method of generating the allocation sequence (eg, computer-generated random numbers), and list of any factors for stratification. To reduce predictability of a random sequence, details of any planned restriction (eg, blocking) should be provided in a separate document that is unavailable to those who enrol participants or assign interventions | Not applicable: non-randomized ward allocation. Sections 5.3.1–5.3.2 (pp. 15–16). |
| Allocation concealment mechanism | 16b | Mechanism of implementing the allocation sequence (eg, central telephone; sequentially numbered, opaque, sealed envelopes), describing any steps to conceal the sequence until interventions are assigned | No random allocation sequence to conceal. Section 5.3.2 (p. 16). |
| Implementation | 16c | Who will generate the allocation sequence, who will enrol participants, and who will assign participants to interventions | Ward allocation: Section 5.3.2 (p. 16). Screening and enrolment: Section 2.4 (pp. 9–10). Consent: Section 4 (pp. 12–13). No random sequence is generated. |
| Blinding (masking) | 17a | Who will be blinded after assignment to interventions (eg, trial participants, care providers, outcome assessors, data analysts), and how | Sections 5.3.1–5.3.2 (pp. 15–16) and 5.4.1 (p. 23): participant blinding cannot be ensured; COPM assessors are not consistently independent and are not blinded to allocation. |
|  | 17b | If blinded, circumstances under which unblinding is permissible, and procedure for revealing a participant’s allocated intervention during the trial | Not applicable: no formal unblinding procedure. Sections 5.3.2 (p. 16) and 5.4.1 (p. 23). |
| **Methods: Data collection, management, and analysis** | | |  |
| Data collection methods | 18a | Plans for assessment and collection of outcome, baseline, and other trial data, including any related processes to promote data quality (eg, duplicate measurements, training of assessors) and a description of study instruments (eg, questionnaires, laboratory tests) along with their reliability and validity, if known. Reference to where data collection forms can be found, if not in the protocol | Sections 5.3.7 (p. 22), 5.4 (pp. 23–30), and 6.1 (pp. 39–40). Collection forms are available on request as described in S3 File, Section 4.9. |
|  | 18b | Plans to promote participant retention and complete follow-up, including list of any outcome data to be collected for participants who discontinue or deviate from intervention protocols | Sections 2.4 (pp. 9–10), 5.5 (pp. 30–31), and 5.7.1 (p. 33): reminders, continued follow-up mailing after nonresponse, assessments after intervention discontinuation when feasible, and participant compensation. |
| Data management | 19 | Plans for data entry, coding, security, and storage, including any related processes to promote data quality (eg, double data entry; range checks for data values). Reference to where details of data management procedures can be found, if not in the protocol | Sections 3.2 (p. 11) and 6–6.3 (pp. 38–40): data entry, verification, pseudonymization, security, storage, and disposal. |
| Statistical methods | 20a | Statistical methods for analysing primary and secondary outcomes. Reference to where other details of the statistical analysis plan can be found, if not in the protocol | Section 5.8 (pp. 34–38). |
|  | 20b | Methods for any additional analyses (eg, subgroup and adjusted analyses) | Section 5.8 (pp. 35–38): adjusted sensitivity analyses, exploratory longitudinal analysis, and secondary analyses. |
|  | 20c | Definition of analysis population relating to protocol non-adherence (eg, as randomised analysis), and any statistical methods to handle missing data (eg, multiple imputation) | Section 5.8 (pp. 37–38): analysis population, multiple imputation, complete-case sensitivity analysis, longitudinal missing-data handling, and available-observation secondary analyses. |
| **Methods: Monitoring** | | |  |
| Data monitoring | 21a | Composition of data monitoring committee (DMC); summary of its role and reporting structure; statement of whether it is independent from the sponsor and competing interests; and reference to where further details about its charter can be found, if not in the protocol. Alternatively, an explanation of why a DMC is not needed | Section 5.6 (p. 32): principal investigator monitoring and rationale for no independent DMC. |
|  | 21b | Description of any interim analyses and stopping guidelines, including who will have access to these interim results and make the final decision to terminate the trial | Exploratory interim analyses have been conducted for conference presentation, but have not been used to stop the trial or change the target sample size or intervention. No efficacy-based stopping rules are planned. Criteria for discontinuing individual participation are described in Section 5.5 (pp. 30–31). |
| Harms | 22 | Plans for collecting, assessing, reporting, and managing solicited and spontaneously reported adverse events and other unintended effects of trial interventions or trial conduct | Section 5.6 (pp. 31–33); Section 5.7.2 (p. 34). |
| Auditing | 23 | Frequency and procedures for auditing trial conduct, if any, and whether the process will be independent from investigators and the sponsor | No independent audit is planned. Routine study oversight and data verification are described in Sections 5.6 (p. 32) and 6.1 (pp. 39–40); see also S3 File, Section 22. |
| Ethics and dissemination | | |  |
| Research ethics approval | 24 | Plans for seeking research ethics committee/institutional review board (REC/IRB) approval | Section 5.1 (p. 14); extension approval in Section 5.3.4 (p. 20). |
| Protocol amendments | 25 | Plans for communicating important protocol modifications (eg, changes to eligibility criteria, outcomes, analyses) to relevant parties (eg, investigators, REC/IRBs, trial participants, trial registries, journals, regulators) | Protocol amendments will be communicated to the institutional ethics committee and the head of the institution before implementation, shared with study staff, and reflected in the UMIN registry as applicable. Participants will be informed and re-consented when necessary (S2 File, Section 19; S3 File, Sections 8 and 19; manuscript, Section 4, pp. 12–13). |
| Consent or assent | 26a | Who will obtain informed consent or assent from potential trial participants or authorised surrogates, and how (see Item 32) | Section 4 (pp. 12–13); Section 2.4 (pp. 9–10). Participant information, consent, and withdrawal forms: S4 File (Japanese) and S5 File (English translation); see Item 32 of this SPIRIT checklist. |
|  | 26b | Additional consent provisions for collection and use of participant data and biological specimens in ancillary studies, if applicable | Section 6.2 (p. 40): renewed consent and ethics approval before future data use. S4 and S5 Files, Section 6. No biological specimens are collected for this study; see Item 33 of this SPIRIT checklist. |
| Confidentiality | 27 | How personal information about potential and enrolled participants will be collected, shared, and maintained in order to protect confidentiality before, during, and after the trial | Sections 6–6.3 (pp. 38–40). |
| Declaration of interests | 28 | Financial and other competing interests for principal investigators for the overall trial and each study site | Competing interests statement in the submission system. |
| Access to data | 29 | Statement of who will have access to the final trial dataset, and disclosure of contractual agreements that limit such access for investigators | Sections 6 and 6.1 (pp. 38–40); S3 File, Section 7.3 (p. 11). Study co-investigators will have access to the pseudonymized final analysis dataset in accordance with the ethics-approved protocol. No contractual agreements restrict investigators’ access to the dataset. |
| Ancillary and post-trial care | 30 | Provisions, if any, for ancillary and post-trial care, and for compensation to those who suffer harm from trial participation | Section 5.6 (pp. 31–32): safety procedures and injury compensation; Section 5.3.6 (p. 22): post-study materials for the control group. |
| Dissemination policy | 31a | Plans for investigators and sponsor to communicate trial results to participants, healthcare professionals, the public, and other relevant groups (eg, via publication, reporting in results databases, or other data sharing arrangements), including any publication restrictions | Section 6 (p. 39). Study findings will be disseminated through academic conferences and peer-reviewed publications, regardless of the direction or magnitude of the findings. No publication restrictions are imposed by the funders. |
|  | 31b | Authorship eligibility guidelines and any intended use of professional writers | Authorship is based on contributions to the study, drafting or critical revision of the manuscript, approval of the final version, and accountability for the work. Author contributions are reported in the submission system. No professional medical writers have been engaged. |
|  | 31c | Plans, if any, for granting public access to the full protocol, participant-level dataset, and statistical code | The full study protocol is provided in S2 and S3 Files (Section 10, p. 52). Plans for public access to participant-level data and statistical code have not yet been determined. |
| Appendices | | |  |
| Informed consent materials | 32 | Model consent form and other related documentation given to participants and authorised surrogates | S4 File (original Japanese) and S5 File (English translation): participant information sheet, consent form, and withdrawal form. Manuscript Section 4 (pp. 12–13) and Section 10 (pp. 52–53). |
| Biological specimens | 33 | Plans for collection, laboratory evaluation, and storage of biological specimens for genetic or molecular analysis in the current trial and for future use in ancillary studies, if applicable | Not applicable. No biological specimens are collected, stored, or analysed for this study. |

*It is strongly recommended that this checklist be read in conjunction with the SPIRIT 2013 Explanation & Elaboration for important clarification on the items. Amendments to the protocol should be tracked and dated. The SPIRIT checklist is copyrighted by the SPIRIT Group under the Creative Commons “[Attribution-NonCommercial-NoDerivs 3.0 Unported](http://www.creativecommons.org/licenses/by-nc-nd/3.0/)” license.
