# Supplementary material for: Study protocol for a non-randomized trial of community participation support for people with Acquired Brain Injury who have ceased driving in Japan
Source: PLoS One. 2026 Nov 10;21(11):e0362487. doi: 10.1371/journal.pone.0362487 (PMC13750569; doi:10.1371/journal.pone.0362487)
Supplement: Supplementary file 2 [file pone.0362487.s002.docx]

**S2 File. Ethics-approved protocol (original Japanese version)**

**研究計画書**

**＜研究課題名＞**

後天性脳損傷後の自動車運転中断者に対しての地域社会参加支援の効果

- 日本における非ランダム化研究 -

**＜研究機関＞**

中伊豆リハビリテーションセンター

**＜研究責任者＞**

作業療法士・那須識徳

**〈分担研究者〉**

**中伊豆リハビリテーションセンター**

生田純一、加納彰

**北海道千歳リハビリテーション大学**

佐々木努

**東京都立大学**

石橋裕

**兵庫医科大学**

小林隆司

**<研究協力者>**

**当事者参加者**

石黒治美

目次

略語及び用語の定義

1. 研究の実施体制と著者の役割

2. 研究期間

3. 研究の目的及び意義

4. 研究の方法

5. 研究対象者の選定方針

6. 研究対象者数、試料・情報の種類及び実施場所

7. 個人情報の取扱い

8. インフォームド・コンセントの手続及び方法

9. 代諾者からインフォームド・コンセントを受ける場合の手続き及び方法

10. インフォームド・アセントを得る場合の手続

11. 研究対象者から取得された試料・情報の将来の活用

12. 試料・情報の保管及び廃棄

13. 研究対象者に生じる負担、リスク及び利益等

14. 研究対象者等及びその関係者からの相談等への対応

15. 研究実施後における医療の提供に関する対応

16. 有害事象発生時の取扱い

17. 当該研究によって生じた健康被害に対する補償

1８. 研究対象者に係る研究結果の取扱い

19. 研究機関の長への報告内容及び方法

20. 研究に関する業務の委託

21. 本研究実施に係る利益相反

22. モニタリング・監査

23. 参考文献

**1. 研究の実施体制**

**１-１．研究実施体制：単施設研究**

研究責任者　中伊豆リハビリテーションセンター　那須 識徳

研究分担者　中伊豆リハビリテーションセンター　生田 純一

　　　　　　　　　　　　　　　　　　　　　　　加納 彰

研究分担者　東京都立大学　　　　　　　　　　　石橋 裕

研究分担者　兵庫医科大学　　　　　　　　　　　小林 隆司

**１-２．研究における役割と責任**

　・責任著者　：那須 識徳

　・連絡著者　：那須 識徳

　・研究の発案：那須 識徳

　・研究デザイン作成：那須 識徳、生田 純一、加納 彰、石橋 裕、小林 隆司

　・データ収集：全職員

　　３階病棟　評価者/介入者：作業療法科全職員

　　２階病棟　評価者/介入者：作業療法科全職員

　　※１評価者・介入者は全職員とする。ただし、情報バイアスをさけるために、評価者と介入者は異なる人物とし、評価者は研究分担者を含めないものとする。

　　　　また、研究責任者は確証バイアスを考慮し評価には加わらないものとする。なお単一施設での実施という研究実施の都合上、介入は研究責任者も行う予定である。（※研究責任者が部署移動に伴い、介入は行わないものとする）

※２倫理的配慮として、感染対策のため各病棟をまたいだ支援は行わない

・データ解析：那須 識徳

・データ解釈：那須 識徳、生田 純一、石橋 裕、小林 隆司

**１-３．研究時の緊急連絡先**

・中伊豆リハビリテーションセンター

・福祉部　さわらび（内［非公開］）

・那須識徳（なす さとのり）

・TEL : ［非公開］

・e-Mail ; sa-nasu@janrc.or.jp

**2. 研究期間**

2024年9月1日～2027年3月31日

**3. 研究の目的及び意義（概要）**

**３-１．背景**

近年、日本国内では高齢化が進み高齢化率は29％と過去最高となった。高齢化の進展に伴い、脳卒中を発症する高齢者が増加していることが知られており、脳卒中は65歳以上の日本人の死因の第3位から第5位を占めている。脳卒中になると、身体機能障害や認知機能障害、知覚障害や心理的な障害など様々な症状が合併することが知られており、日常生活に様々な問題を引き起こす。そのような背景のもと、近年、リハビリテーション医療分野では、脳卒中者の自動車運転支援に注目が寄せられている。国内で脳卒中患者525名に行った自動車運転再開の実態調査では、退院時の自動車運転再開志望者が約70％いた^1）^とされているが、その再開率は３割～５割程度となっている。先行研究によれば、脳損傷者が運転を再開できないと、地域社会との繋がりが途切れたり、長期介護施設入所のリスクが高まったり、抑うつ傾向になることが知られている。そのため、なんらかの影響により運転を中断せざるを得ない方に対して運転中断支援を行っていくことは重要な課題であるが、その介入方法についての枠組みを示した研究は少なく、介入研究も十分に行われていない。

我々が運転中断者に行った調査では、運転再開者と運転中断者では、本人がやりたいこと、する必要があること、健康関連QOL、活動への参加状況に差があることが示された^2）^。つまり、運転が行えずに退院すると、本人がやりたいことや、する必要があることが行えず、健康関連QOLなどに影響を生じさせる可能性がある。しかし、同データを利用したサブ解析にて、運転中断群でも「自分で運転を辞める」と決めた群は、「医師や家族から促されて運転を辞めた群」に比べ、自分の行いたいことができるという結果が示された^３）^。さらに、運転を中断した場合でも地域で自分の行いたいことができている者はそうでないものに比べ、その後の運転再開率が高いことも分かった。これらの研究結果は、脳損傷後の運転中断支援の有効性を検証するために有益な情報になりうると考える。

**３-２．介入をデザインするための背景理論**

我々は、後天性脳損傷後の運転中断者の質的な研究を統合して、作業療法分野の介入の枠組みである人、作業、環境モデルを使用して介入の枠組みである中範囲理論を構築した^4）^。

「人」は当事者だけでなく、運転中断の当事者を支援する家族や近親者を含んでいる。後天性脳損傷後に運転を中断すると、地域の移動が困難となることが多い。そのため、家族などの近親者に地域の移動の協力を得ることが多いという。しかし、頻回な協力依頼は当事者だけでなく家族の負担になりうるため、運転中断後の地域移動支援には家族などの協力者への関わりも介入時に検討することが重要であることを示している。

「作業」とは当事者にとって運転という作業がどのような意味や役割、価値をもたらしていたかを示す。運転中断により困難になっている活動を探り、中断後にも、その活動を継続するための方法や手段を検討する必要がある。

「環境」は利用可能な公共資源や代替交通機器、各種サービス、当事者を支援する人などを示す。運転中断の当事者がおかれている環境で地域移動に利用できるものは何か、個別に検討していくことで、運転中断者に対する支援は、より充実したものとなる可能性がある。

運転を中断した場合、多くの当事者は、代替資源（代替交通、移動サービス）が乏しいことを指摘する。しかし、活用できる資源は少なくとも、入院中から運転中断後の生活を想定することで運転中断後の漠然とした不安を明確にし、必要な情報提供や代替移動機器の練習を行い、対応方法を検討できれば、退院後の地域社会との繋がりを促し、活動量の低下を防ぐ可能性はある。さらに、運転を中断したとしても、地域でやりたいことを継続することが運転再開の可能性を高めることを仮定すれば、運転中断者は、運転を諦めるために代替手段などを使用するのではなく、運転再開のために前向きに運転中断後の生活を考えられるのではないかと考えている。本プロジェクトは後天性脳損傷後( Acquired Brain Injury; ABI )に運転を中断したとしても、地域・社会との繋がりを作る一端を担うことを期待している

**３-３．研究の目的**

　病気や怪我の後に運転を中断せざるを得ない方に対して、「人」「作業」「環境」の理論を用いて構築した、「運転中断後の当事者と家族の経験モデル」を使用し、当事者参加型の**運転中断後の地域生活支援プログラム：Community Life Support Program** for Individuals with Acquired Brain Injury after Driving Cessation **(CLASP-AID)**を構築し、その介入効果を検討することを目的とする。なおCLASP-AIDのトライアルに関しては，北海道作業療法士協会の依頼によって2024年3月30日に実施した「自動車運転を中止した脳卒中当事者に対する地域移動支援」の中の事例報告において実施済みである。トライアルを行う中で対象者に不利益や負担は生じておらず、一定の効果が得られたため、本介入では先行した支援を改変した上で、非ランダム化比較試験として計画した。

具体的には、主要アウトカムとして、「運転中断後に支障をきたす可能性のある活動」をCOPMにて確認し、その満足度と実行度を確認する予定である。副次的なアウトカムでは、「運転中断の準備性」、「抑うつ傾向」や「生活の質」、退院後の「環境因子」について確認し、介入群とコントロール群で効果の差の比較を行う予定である。

**【研究仮説】**

介入群は、対象群に比べ自動車運転中断に対する準備ができており、退院後に必要な活動に対する実行度や満足度が高い傾向にある。

**４. 研究の方法**

**４-１. 研究デザイン**　非ランダム化比較試験

介入の有無　　：有（プログラム実施群：A群、コントロール群：B群）

対照群の有無　：有

侵襲の有無　　：無

検証するレベル：非ランダム化比較試験

研究の規模　　：単施設研究

研究結果の広告利用の可能性：有

研究結果はセンター内広報として広く、一般の方々に認知できるよう広報を行う

**４-２. 研究の方法**

１）割付方法：非ランダム化比較試験　non-randomized controlled trial

クラスター割り付け本研究は単一の施設（回復期リハビリテーション病院）にて行

われた。そのため完全な無作為化や盲検化は困難であった。コントロール群が介入

に暴露される可能性を最大限に減らすために、病棟ごとに介入群とコントロール群

に分けて実施することとした。なお、割り付けの隠蔽については、いずれの郡に対

しても「AとBの介入があり、そのどちらかの介入を受けて頂きます」と伝えた

上で、具体的にどちらの郡に割り付けされたがわからないよう最大限の配慮を行な

った。

**２階病棟**：コントロール群、**３階病棟**：介入群

２）構築したプログラムCLASP-AIDの詳細

① 運転中断支援プログラム（当事者４回、家族×１回）

※　１回目の講義のみ介入群もコントロール群も実施する。

**[当事者]**

　 a 研究機関における運転支援について

b 運転がすぐに再開できなかった場合に考えておくこと

　 c 自動車運転の代わりの交通手段について

　 d 運転中断後の具体的な生活について考える

**[家族]**

　 e 家族や近親者が支援するポイント

② 地域のサービスや公共交通などの情報整理と提供

近隣地区のパンフレットの作成と移動資源の提供：伊豆市はパンフレット作製済

③ シニアカーや電動車いすなどの代替交通機器使用の評価と介入

３）介入期間　202４年９月１日～202７年の３月31日

４）介入時期

介入の開始時期は、自動車運転評価終了後、医師とOTでのカンファレンスを行った後に、本人及び家族や近親者に説明を行い実施する。なお、CLASP-AIDのプログラム①は運転評価開始時に実施するため、A群もB群も実施することとする。その後、介入群はプログラムの②～④を１週間程度かけて実施し、最後にご家族に対して⑤のプログラムを実施し終了する予定である。

　５）介入内容

作成した運転中断支援プログラム（プログラム②）に沿って、当事者と家族へ講義を行う。具体的には運転中断後に支障をきたす可能性のある活動の実行度と満足度確認した上で、それらの活動の実行度と満足度を確認する。その後、支障をきたすことが予想される活動を継続するために、当事者に必要な地域移動資源の提示（プログラム③）、代替移動機器の練習（プログラム④）を行う予定である。

なお、介入群（A群）には構築したプログラムを実施し、非介入群（B群）には従来の支援を実施する予定である。A群とB群の違いは、教育プログラムの有無であり、非介入群においても、代替移動機器や代替移動支援サービスはこれまでの支援と同様に行われる予定である。また、B群に対しては、希望が聞かれた場合A群で実施した教育プログラムの資料を研究終了後に配布する予定である。

６）介 入 者

研究実施機関で自動車運転評価を担当する運転係の作業療法士とした。なお

CLASP-AIDの実施者に対しては、実施方法についての説明を事前に行った上で、

実施することとした。

**４-３. 評価の方法**

評価のベースラインは、担当医と担当OTのカンファレンスにて運転再開にリスクを伴うと判断を行った日とする。ベースラインから１週間以内に初回の評価（主要アウトカム、副次的アウトカム）を行う。入院中および通院中の評価は信頼性と妥当性が確認されている評価指標を使用する予定である。その後、連絡が可能で、かつ評価への回答が可能であり、本人の協力意思が得られた研究対象者に対して、退院後1か月、3か月、6か月に質問紙による追跡評価を実施する。これらの追跡評価は、退院後の経時的変化を探索的に確認するために用いる。

**４-４．主要アウトカム**

① COPM：Canadian Occupational Performance Measure

COPMは、職業パフォーマンスにおける自己認識の問題を特定し評価すること

を目的とした半構造化された面接である。本研究では、COPMを運転中断の文

脈で適用し、運転中断に関連する作業遂行度を測定する。面接中、参加者には、

日常生活、地域移動、社会参加など、運転中断の結果として困難を感じている

活動や役割を特定するよう求める。参加者はその後、最大5つの作業遂行上の

課題を選択し、各項目の遂行度は１〜10点の尺度で評価する。スコアが高い

ほど、より良い遂行度を示す。主要評価時点は介入直後とし、介入前のCOPM

遂行度を調整した上で、介入直後のCOPM遂行度を群間比較する。

**４-５．副次的アウトカム**

① 運転中断準備状況の評価

日本語版Assessment of readiness for mobility transition：ARMT-J

ARMTは、加齢により移動を変更する必要がある場合の、当事者の感情や態度

の準備状況を確認するための質問紙である。ARMT-J は11 項目で構成され

おり、AA：予測される不安、PB：認識される負担感、AS：不利な状況以上の

３つの下位尺度に分類することができる。各項目は「1点：全くそう思わない」

～「5点：非常にそう思う」の5段階のリッカートスケールとなっており、得

点の解釈は下位尺度の平均得点でなされる。

② QOLの評価：SF12（人）

Medical Outcomes Study 12-Item Short-Form Health Survey version 2

SF-12は健康関連QOLを評価するための信頼性・妥当性を持つ評価尺度であ

り、身体機能、日常役割機能（身体）、体の痛み、全体的健康観、活力、社会

生活機能、日常役割機能（精神）、心の健康の8つの下位尺度が算出可能な質

問紙である。8つの下位尺度はさらに上位の概念として、身体的側面のQOL

（Physical component summary：PCS）、精神的側面のQOL（Mental

component summary：MCS）、役割／社会的側面のQOL(Role-social

component score：RCS）の3コンポーネントで得点化するサマリースコア

の算出が可能である。得点が高いほど健康関連QOLは高いとされ、本研究で

は、PCS、MCS、RCSスコアを解析対象に用いた。

③ 抑うつ傾向の評価（人）Geriatric depression scale 15 ：GDS-15

うつのスクリーニング検査として使用されている検査であり、信頼性と妥当性　も確認されている評価である。１５問の質問から成り立っている簡便な検査であり「はい」か「いいえ」で答える形式なので、答えやすく採点が容易である。５点以上がうつ傾向、10点以上がうつ状態とされている。

④ 環境面の評価（環境）

　 Comprehensive Environmental Questionnaire for the Elderly ：CEQ

CEQは在宅高齢者のQOLを高めることを目的として、信頼性・妥当性が確認

され、安心生活環境（6項目）、相互交流環境（6項目）、家族環境（2項目）

の3因子合計14項目で構成される。「まったくない（1点）」から「十分あ

る（4点）」の4件法で評定し単純加算して因子別スコア、そうスコアを算出

し、得点が高いほど満足した環境にあると解釈することができる。

⑤ Life Space Assessment：LSA（前向き調査のみ）

LSA は、身体活動を生活空間の概念でとらえ、居室、居宅内、居住空間のごく

近くの空間（庭やアパートの敷地内）、自宅近隣、町内、町外の各生活範囲に

おける活動の有無、頻度、自立度を調査した結果から得点化（0～120 点）す

る尺度である．調査時間は 10 分程度と簡便であり、信頼性および妥当性が検

証された臨床的有用性の高い評価指標である。

　⑥ 電動モビリティー機器の評価について

　　 電動モビリティー機器の練習は、研究実施機関にあるモビリティー機器のコー

スにて評価を行う予定であるが、最終的に地域で使用する場合は、専門業者の

支援と評価を受けてもらい機器の利用が可能か判断を行ってもらう予定である。

**４-6．その他の一般情報**

1. 年齢
2. 性別
3. 疾患名
4. 発症日からの日数
5. 移動支援者の有無
6. 運動麻痺の程度(Fugl Meyer Assessment : FMA)
7. 認知機能の程度（Mini Mental State Examination : MMSE）
8. 日常生活の自立度(Functional Independence Measure : FIM)

**4-7．安全性評価項目**：検査前と後に血圧・脈拍・血中酸素飽和度を測定する

**4-8．研究の中止及びその方法**

中止基準

- 研究対象者から研究参加の辞退の申し出や同意の撤回があった場合
- 登録後に選択基準を満たさないことが判明した場合
- 原疾患の悪化のため、評価の継続が好ましくないと判断された場合
- 合併症の増悪により評価の継続が困難な場合

脱落基準

- 研究対象者が死亡した場合

**4-9．統計学的手法**

　すべてのデータはExcelに入力し，R version 4.5.2（R Foundation for Statistical Computing, Vienna, Austria）を用いて解析を行う。連続変数は平均値および標準偏差で，カテゴリ変数は度数および割合で要約する。連続変数の正規性はShapiro–Wilk検定により評価する。

　主要解析ではCOPMの遂行度（performance）を主要アウトカムとする。介入後のCOPM遂行度の群間差については，介入前のスコアを共変量とした共分散分析（ANCOVA）により検討する。さらに，介入前から介入後および退院後1か月，3か月，6か月までの経時的変化については，群，時間，および群×時間の交互作用を固定効果とした混合効果モデルを用いて解析する。なお，本研究は病棟単位で割付を行っており，対象となるクラスターが2病棟に限られるため，クラスター効果の統計学的な調整は行わず，結果は探索的に解釈する。なお，病棟間の背景因子の違いが結果に影響を与える可能性がある点には留意する。

　副次アウトカムについては記述統計を中心に解析し，変化の傾向を補助的に検討する。各時点における群間比較については，適切な記述統計量を用いて示す。本研究はパイロット研究であるため，副次アウトカムに対する形式的な仮説検定は主目的とはしない。また，ARMT-Jとベースライン特性との関連については，ピアソンの相関係数を用いて探索的に検討する。主要解析における有意水準はp < 0.05とする。欠測値が認められた場合には，解析に先立ち多重代入法の適用を検討する。

　質問紙による評価については標準化されたデータ収集用紙を使用し，必要に応じて責任著者に問い合わせることで入手可能とする。

　解析は，プロトコル逸脱の有無にかかわらず，少なくとも1時点のアウトカムデータが得られた対象者を対象とした修正intention-to-treat原則に基づいて実施する。

**５．研究対象者の選定方針**

**５-1．対象**

日本の静岡県にある回復期リハビリテーションセンターで入院あるいは通院しており、自動車運転評価を受け、運転が再開できなかった後天性脳損傷患者を対象とする。対象年齢は55歳以上で、設定の根拠はARMT-Jの研究における対象者の設定に合わせている。

**５-2．選択基準**

１）本研究の参加にあたり、十分な説明を受けた後に、十分な理解の上に、本人の自由意志による文書同意が得られた患者。

２）同意取得時に５５歳以上の患者。

３）普通自動車運転免許で運転を中断したもの

**５-３．除外基準**

１）てんかんや統合失調症、認知症など公安委員会で定められた、運転再開の

欠格事由者は除外する。

２）MMSEが24点以下の者で明らかな認知機能障害があるもの

３）失語症の患者で検査の理解が十分にできないもの

**５-４．設定根拠**： 研究対象者の安全性確保及び研究基準の設定のために設定した

**５-５．選定の流れ**：4-2. 研究の方法、１）割付方法に準ずる

**６．研究対象者数、試料・情報の種類及び実施場所**

**６-1．研究対象者数**

　本研究は、病棟への入院に基づいて割付を行う単施設のパイロット・探索的研究であるため、研究対象者数は、研究実施施設における適格患者数と研究期間中の実施可能性に基づいて設定した。介入群と対照群の群間差および介入前後のCOPM遂行度の関連について、信頼できる事前推定値が得られなかったため、予定する共分散分析に対する形式的な検出力計算は実施しなかった。

主要解析では、介入直後のCOPM遂行度を主要評価時点とし、介入前のCOPM遂行度を共変量とした共分散分析により群間差を推定する。主要評価項目を解析可能な対象者数として30例を予定し、評価の欠測や脱落を考慮して、最大40例まで登録する可能性がある。病棟への入院によって群が決まるため、各群を同数にすることは前提としない。

本研究の対象者数は、介入効果の確定的な検証や病棟単位のクラスター効果を統計学的に調整することを目的としたものではない。主要解析では、調整後の群間差と95％信頼区間を示し、効果の大きさ、ばらつき、登録率、追跡率およびデータの完全性を、将来の検証的研究の設計と対象者数算定に用いる。**６-２．資料・情報の種類**

　研究実施中に得られる資料は紙媒体にて記録し、記録後は研究実施機関内で保有しているPCに記録する予定である。

**６-３．実施場所**

CLASP-AIDおよび関連する評価は、農協共済中伊豆リハビリテーションセンターOT室にて実施する。運転の代替手段として代替モビリティー機器が必要になった場合は、院内のモビリティーコースを利用して評価・介入する予定である。代替モビリティー機器の評価はWCTにて確認する予定である。

**７．個人情報等の取扱い**

**７-１．匿名化の有無：あり**

**７-２．匿名化の方法：連結可能匿名化**

**７-３．情報の管理・保管**

　・入手したデータは研究のみに使用し守秘義務を厳守し、当院倫理審査委員会管理の元、厳重に保管する（書字室内の鍵付き保管庫にて保管。）

　・学会や学術雑誌における研究発表では、面接データを統計的な処理を行なった上で公開するので、個人が特定されることはない。

　・部外の分担研究者とデータの交換を行なう際には、個人が特定される情報を暗号化し、個人情報の漏洩にならないよう配慮を行なう。

**７-４．個人情報の開示**

　・研究対象者から個人情報の開示を求められた場合他の研究対象（協力）者等の個

人情報の保護及び本研究の独創性の確保に支障がない範囲内で、研究計画及び研

究の方法に関する資料を提示する。その場合は、説明文書末尾の本研究に関して

の問い合わせ先に連絡頂くよう、説明文書にて提示する。

**８．インフォームド・コンセントの手続き及び方法**

**８-１．文書IC**

・研究責任者及び研究分担者は、倫理審査委員会で承認の得られた同意説明文書を研究対象者に渡し、文書及び口頭による十分な説明を行い、研究対象者の自由意思による同意を文書で取得する。研究責任者及び研究分担者は、研究対象者の同意に影響を及ぼす情報が得られたときや、研究対象者の同意に影響を及ぼすような実施計画等の変更が行われるときは、速やかに研究対象者に情報提供し、研究に参加するか否かについて研究対象者の意思を予め確認するとともに、事前に倫理審査委員会の承認を得て同意説明文書等の改訂を行い、研究対象者の再同意を得ることとする。同意説明文書には、指針で必要とされる内容を含むものとする。

**８-２．口頭IC及びその記録**

・研究責任者及び研究分担者は、研究対象者に対し、研究の参加等について口頭による十分な説明を行う。研究対象者の自由意思による同意の意思を口頭で確認し、同意の日時・説明方法・説明者・同意事項等についてカルテに記録する。

研究責任者及び研究分担者は、研究対象者の同意に影響を及ぼす情報が得られたときや、研究対象者の同意に影響を及ぼすような実施計画等の変更が行われるときは、速やかに研究対象者に情報提供し、研究に参加するか否かについて研究対象者の意思を予め確認するとともに、その旨、カルテに記録する。

ICへ含む内容

・研究の概要（目的および方法と期間）

・研究参加は任意であること

・研究に参加することに同意をしない場合でも不利益を受けないこと

・研究機関の名称並びに研究機関の長及び研究責任者の氏名

・研究計画書及び研究の方法に関する資料を入手又は閲覧できる旨

・個人情報の取り扱いと開示に係る手続

・得られたデータの利用範囲

・研究に参加することで得られる利益と不利益

・研究の当該領域・社会に対する貢献

・研究の中止と拒否について

・研究対象者及びその関係者からの相談窓口

※1 本研究で考えられる研究対象者の負担は、「1３-2． 予測されるリスク」に

記す通りである。

※２研究責任者及び研究分担者は、研究対象者の同意に影響を及ぼす情報が得ら

れたときや、研究対象者の同意に影響を及ぼすような実施計画等の変更が行われるときは、速やかに研究対象者に情報提供し、研究に参加するか否かについて研究対象者の意思を予め確認するとともに、事前に倫理審査委員会の承認を得て同意説明文書等の改訂を行い、研究対象者の再同意を得ることとする。同意説明文書には、指針で必要とされる内容を含むものとする。

**9．代諾者等からインフォームド・コンセントを受ける場合の手続及び方法**

・代諾とは本人の代わりに承諾することであり、本研究では明らかな認知機能の低

下や失語症を生じた患者を対象とはしない。そのため、本研究では該当しない。

**1０．インフォームド・アセントを得る場合の手続**

・小児を対象とした研究ではないので該当しない

**１１．研究対象者から取得された試料・情報の将来の活用**

・研究対象者から取得された情報について、研究対象者から同意を受ける時点では特定されない将来の研究のために用いられる可能性として、より規模の大きな研究に発展した場合に今回の研究で得た情報を、将来の研究に使用することが予想される。その場合の目的の概要を研究対象者に説明を行う。

本研究終了後、試料を将来の研究に使用することに同意されている場合は、当院倫理委員会の責任のもとに保管する。新たに企画された研究を実施する前には当該の倫理委員会（臨床試験審査委員会）に申請し審査を受ける。同意後も、提供者本人（あるいは代諾者）からの同意撤回があれば本人の意向に沿って試験に伴う試料・情報を廃棄する。

**1２．試料・情報の保管及び廃棄**

・本研究終了時には、試料・情報を長期保存して将来の新たな研究に使用することに同意している場合を除き、原則として研究の中止または終了後少なくとも5年間、あるいは研究結果発表後3年が経過した日までの間のどちらか遅い期日まで保存する。紙資料の廃棄はシュレッダーで行い、USBなどの電子媒体はデータを完全消去し破壊することで廃棄することとする。

**１３．研究対象者に生じる負担、リスク及び利益等**

**１３-1．予測される利益**

・本研究では介入研究を行うが、介入効果を示した根拠はこれまでにない。そのため、参加することによる被験者に直接の利益は生じない。研究成果により将来の医療の進歩に貢献できる可能性がある。また、事後のアンケート調査に関しては全３回の協力をもって、クオカード1,000円分をアンケート協力者に送付することとする。

**1３-2．研究対象者に生じる負担、予測されるリスク**

・患者へのリスク負担：介入群はこれまで行ってこなかった運転中断支援のための教育プログラムが追加される。プログラム参加への負担は懸念されるものの、これまでにも運転中断後のフォローは各訓練士に任せて実施していた経緯があることから倫理的な問題はないものと考える。ただし、プログラム参加において疲労などが生じた場合は、休憩の実施や研究の中止を検討する。

・予測されるリスク：本研究では電動車いすやシニアカーの利用を検討している。

モビリティーコースでは転倒の危険性もあるために、訓練士は必ず実施者の横に

立ち、いつでもサポートできるように対応する。また、事故防止に努めるため、実車評価はモビリティーコース内及び、車両が走行していない運転コースで実施することとし、段差や傾斜は事前の減速を訓練士が管理することで、転倒予防に努めることとする。さらに希望者にはヘルメットの着用を行うこととする。また、他の車両が並走している場合は、運転評価を行わないこととする。

**1３-3．事故が起きたときの対応について**

・屋外において事故が起きた時は、まず持参している携帯電話にてセンターに電話

を行い、応援職員を呼んで対応する。その後の事故処理は当院の安全管理委員会

のインシデント対応に準ずるものとする。

1**４．研究対象者等及びその関係者からの相談等への対応**

・相談への対応は、中伊豆リハビリテーションセンターの同意書に基づき、対応を

行う。

**１５．研究実施後における医療の提供に関する対応**：該当しない

**１６．有害事象発生時の取扱い**：当センターでは、事故が起きた時のために保険に加

入している。そのため、万が一事故が起きた場合は、当センターで加入してい

る保険にて対応することにする。屋外において事故が起きた時は、まず持参し

ている携帯電話にてセンターに電話を行い、応援職員を呼んで対応する。その

後の事故処理は当院の安全管理委員会のインシデント対応に準ずるものとす

る。

**１７．当該研究によって生じた健康被害に対する補償**：該当しない

**１８．研究対象者に係る研究結果の取扱い**：該当しない

**１９．研究機関の長への報告内容及び方法**

・研究責任者は、研究計画書を変更して研究を実施しようとする場合、予め研究計画書を変更し、倫理審査委員会事務局を介し、研究機関の長へ報告する。その他、研究機関の長へ報告が必要な事項も、原則倫理審査委員会事務局を介して行う。

**2０．研究に関する業務の委託**

「本研究の実施において、外部へ委託する業務は特にない」

**2１．本研究実施に係る利益相反**：無し

・研究の資金源：本研究は2023年トヨタ財団の研究助成を得て実施している。

**2２．モニタリング・監査**：該当しない

**2３．参考文献**

１）武原格，他：脳卒中患者の自動車運転再開についての実態調査. 日本交通科学協議会誌 9(1)：51-55，2009．

２）那須識徳, 他：山間地域における脳卒中後の運転再開群と運転中断群の作業参加状況の違い：アンケートによる調査研究. 作業行動研究, 26(3), 139-146，2022．

３）那須識徳, 他：後天性脳損傷後の自動車運転中断の意思決定要因の違いが作業参加に及ぼす影響.-アンケートによる予備的調査研究- 作業療法，in press．

4）那須識徳,他：後天性脳損傷後に自動車運転を中断した当事者と家族の経験─ 質的研究 Meta-study を用いて─. 作業療法, 41(6), 663-675.

５）Stowe, J. Mobile aging: A randomized trial of mobility transition counseling for high-risk older adults (Doctoral dissertation, University of Missouri--Columbia). 2014.
