# Supplementary material for: Study protocol for a non-randomized trial of community participation support for people with Acquired Brain Injury who have ceased driving in Japan
Source: PLoS One. 2026 Nov 10;21(11):e0362487. doi: 10.1371/journal.pone.0362487 (PMC13750569; doi:10.1371/journal.pone.0362487)
Supplement: Supplementary file 3 [file pone.0362487.s003.docx]

**S3 File. Ethics-Approved Study Protocol (Complete English Translation)**

Study Protocol

**Study title**

Effects of Community Participation Support for Individuals Who Have Ceased Driving After Acquired Brain Injury: A Non-Randomized Study in Japan

**Research institution**

Nakaizu Rehabilitation Center

**Principal investigator**

Satonori Nasu, Occupational Therapist

**Co-investigators**

- Nakaizu Rehabilitation Center: Junichi Ikuta; Akira Kano
- Hokkaido Chitose College of Rehabilitation: Tsutomu Sasaki
- Tokyo Metropolitan University: Yu Ishibashi
- Hyogo Medical University: Ryuji Kobayashi

**Research collaborator / person with lived experience**

Harumi Ishiguro

Contents

Definitions of abbreviations and terms

1. Study organization and author roles
2. Study period
3. Purpose and significance of the study
4. Study methods
5. Policy for selecting participants
6. Number of participants, types of materials/information, and study sites
7. Handling of personal information
8. Procedures and methods for informed consent
9. Procedures and methods when informed consent is obtained from a legally authorized representative
10. Procedures for obtaining informed assent
11. Future use of specimens and information obtained from participants
12. Storage and disposal of specimens and information
13. Burdens, risks, and benefits for participants
14. Response to consultations from participants and related persons
15. Provision of medical care after completion of the study
16. Handling of adverse events
17. Compensation for health injury caused by the study
18. Handling of research results pertaining to participants
19. Content and method of reports to the head of the research institution
20. Outsourcing of study-related activities
21. Conflicts of interest related to the study
22. Monitoring and auditing
23. References

Definitions of Abbreviations and Terms

No definitions were listed in the source document.

1. Study Organization

1.1 Study organization

This is a single-center study.

- Principal investigator: Satonori Nasu, Nakaizu Rehabilitation Center
- Co-investigators: Junichi Ikuta and Akira Kano, Nakaizu Rehabilitation Center
- Co-investigator: Yu Ishibashi, Tokyo Metropolitan University
- Co-investigator: Ryuji Kobayashi, Hyogo Medical University

1.2 Roles and responsibilities

- Responsible author: Satonori Nasu
- Corresponding author: Satonori Nasu
- Study conception: Satonori Nasu
- Study design: Satonori Nasu, Junichi Ikuta, Akira Kano, Yu Ishibashi, and Ryuji Kobayashi
- Data collection: All staff members
- Third-floor ward assessors/intervention providers: All occupational therapy staff
- Second-floor ward assessors/intervention providers: All occupational therapy staff
- Data analysis: Satonori Nasu
- Data interpretation: Satonori Nasu, Junichi Ikuta, Yu Ishibashi, and Ryuji Kobayashi

Assessors and intervention providers were originally to include all staff members. To reduce information bias, different individuals were to serve as assessors and intervention providers, and assessors were not to include co-investigators. The principal investigator was not to participate in assessment because of the potential for confirmation bias. Because this is a single-center study, the principal investigator was initially expected to provide the intervention; however, following a departmental transfer, the principal investigator will not provide the intervention. As an infection-control measure, staff will not provide support across wards.

1.3 Emergency contact during the study

- Nakaizu Rehabilitation Center
- Welfare Department, Sawarabi (extension [redacted])
- Satonori Nasu
- Telephone: [redacted]
- Email: sa-nasu@janrc.or.jp

2. Study Period

September 1, 2024 to March 31, 2027.

3. Purpose and Significance of the Study (Overview)

3.1 Background

In recent years, population aging in Japan has progressed, and the proportion of older adults has reached a record high of 29%. The incidence of stroke among older adults is known to increase with population aging, and stroke ranks between the third and fifth leading cause of death among Japanese people aged 65 years or older. Stroke may be accompanied by physical, cognitive, perceptual, psychological, and other impairments, which can cause various problems in daily life. Against this background, support for resuming automobile driving after stroke has recently received attention in rehabilitation medicine. In a Japanese survey of 525 patients with stroke, approximately 70% wished to resume driving at discharge, but only approximately 30%–50% actually resumed driving [1]. Previous studies have reported that failure to resume driving after brain injury may disrupt connections with the community, increase the risk of admission to long-term care facilities, and contribute to depressive symptoms. Supporting people who must cease driving for health-related reasons is therefore important. However, few studies have presented structured frameworks for such support, and intervention research remains insufficient.

In our previous survey of people who had ceased driving, differences were found between those who resumed driving and those who did not in activities they wanted or needed to perform, health-related quality of life, and participation in activities [2]. Thus, discharge without the ability to drive may prevent individuals from performing desired or necessary activities and may adversely affect health-related quality of life. A secondary analysis of the same data showed that, among those who ceased driving, people who personally decided to stop driving were better able to perform desired activities than those who stopped at the urging of a physician or family member [3]. We also found that people who continued to perform desired community activities despite driving cessation were more likely to resume driving later. These findings may provide useful information for evaluating support after driving cessation following brain injury.

3.2 Theoretical basis for intervention design

We integrated qualitative studies concerning people who ceased driving after acquired brain injury and constructed a middle-range theory using the person–occupation–environment model, an intervention framework in occupational therapy [4].

“Person” includes not only the affected individual but also family members and close relatives who provide support. Following driving cessation after acquired brain injury, community mobility often becomes difficult, and individuals frequently rely on family members or other close persons for transportation. Repeated requests for assistance may burden both the individual and the family. It is therefore important that interventions addressing community mobility after driving cessation also consider support for family members and other people who assist with mobility.

“Occupation” refers to the meaning, role, and value that driving had for the individual. Activities made difficult by driving cessation should be identified, and methods and means of continuing these activities should be considered.

“Environment” refers to available public resources, alternative transportation devices, services, and people who provide support. Individually examining the resources available for community mobility in each person’s environment may enrich support after driving cessation.

Many people who cease driving report limited access to alternative resources, including transportation options and mobility services. Even when resources are scarce, anticipating life after driving cessation during hospitalization may help clarify vague concerns, provide necessary information, enable practice with alternative mobility devices, and identify practical solutions. These efforts may promote continued community connections after discharge and prevent reduced activity. If continuing desired community activities increases the possibility of eventually resuming driving, alternative mobility need not represent giving up driving; instead, it may allow people to think positively about life during driving cessation while working toward possible resumption. This project is expected to help people who cease driving after acquired brain injury maintain connections with their communities and society.

3.3 Study purpose

The purpose of this study is to develop and examine the effects of the Community Life Support Program for Individuals with Acquired Brain Injury after Driving Cessation (CLASP-AID), a participatory community-living support program based on the “experience model of individuals and families after driving cessation,” which was constructed using person, occupation, and environment theory. A preliminary CLASP-AID trial was included in a case report entitled “Community mobility support for a person with stroke who ceased automobile driving,” presented on March 30, 2024 at the request of the Hokkaido Association of Occupational Therapists. No disadvantages or burdens occurred during that trial, and some benefit was observed. The previous support was therefore modified and the present intervention was planned as a non-randomized controlled trial.

The primary outcome will be the Canadian Occupational Performance Measure (COPM), which will identify activities likely to be affected by driving cessation and assess performance and satisfaction. Secondary outcomes will assess readiness for driving cessation, depressive symptoms, quality of life, and environmental factors after discharge, and differences between the intervention and control groups will be examined.

**Study hypothesis:** Compared with the control group, the intervention group will demonstrate greater readiness for driving cessation and tend to report higher performance and satisfaction for activities needed after discharge.

4. Study Methods

4.1 Study design

- Design: Non-randomized controlled trial
- Intervention: Yes (program group: Group A; control group: Group B)
- Control group: Yes
- Invasive procedures: No
- Level of evidence to be examined: Non-randomized controlled trial
- Scale: Single-center study
- Potential use of findings for publicity: Yes

The study findings will be publicized through the Center’s communications so that they are broadly accessible to the public.

4.2 Study procedures

1) Allocation

This non-randomized controlled trial uses cluster allocation. The study is conducted at a single facility, a convalescent rehabilitation hospital; therefore, complete randomization and blinding are difficult. To minimize the possibility that the control group will be exposed to the intervention, intervention and control conditions are assigned by ward. To conceal allocation as much as possible, participants in both groups are told that there are interventions A and B and that they will receive one of them, without being told their specific group.

- Second-floor ward: Control group
- Third-floor ward: Intervention group

2) Details of the CLASP-AID program

**Driving cessation support program:** Four sessions for participants and one session for family members. Only the first lecture is provided to both intervention and control groups.

For participants:

- a. Driving support at the study institution
- b. Issues to consider when driving cannot be resumed immediately
- c. Transportation alternatives to automobile driving
- d. Planning concrete daily life after driving cessation

For family members:

- e. Key points for support by family members and close relatives

The intervention also includes organizing and providing information about community services and public transportation, including preparation of pamphlets and information on local mobility resources. A pamphlet has already been prepared for Izu City. Assessment and intervention concerning alternative mobility devices, such as mobility scooters and powered wheelchairs, are also included.

3) Intervention period

September 1, 2024 to March 31, 2027.

4) Timing of the intervention

After completion of the automobile driving assessment, a conference will be held between the physician and occupational therapist. The intervention will then be explained to and implemented with the participant and, where relevant, family members or close relatives. Because CLASP-AID Program 1 is conducted at the beginning of the driving assessment, it will be provided to both Groups A and B. The intervention group will subsequently complete Programs 2–4 over approximately one week, followed by Program 5 for family members.

5) Intervention content

Lectures will be provided to participants and family members in accordance with the driving cessation support program (Program 2). Activities likely to become difficult after driving cessation will be identified and their performance and satisfaction assessed. Participants will then receive information about community mobility resources needed to continue these activities (Program 3) and training in alternative mobility devices (Program 4).

The intervention group (Group A) will receive the structured program, whereas the non-intervention group (Group B) will receive conventional support. The difference between Groups A and B is the presence or absence of the educational program. Alternative mobility devices and alternative mobility support services may also be provided to Group B as part of conventional support. If requested, materials from the educational program provided to Group A will be distributed to Group B after completion of the study.

6) Intervention providers

The intervention will be delivered by occupational therapists on the driving team who are responsible for automobile driving assessment at the study institution. Providers will receive an explanation of the CLASP-AID delivery procedures before implementing the program.

4.3 Assessment procedures

Baseline is defined as the date on which the attending physician and occupational therapist determine at a conference that resumption of driving poses a risk. The initial primary and secondary outcome assessments will be conducted within one week of baseline. During hospitalization and outpatient care, measures with established reliability and validity will be used. Following discharge, questionnaire-based follow-up assessments are scheduled at 1, 3, and 6 months for participants who can be contacted and are able and willing to complete them. These follow-up assessments will be used to explore changes over time.

4.4 Primary outcome

Canadian Occupational Performance Measure (COPM)

The COPM is a semi-structured interview designed to identify and evaluate self-perceived problems in occupational performance. In this study, it is applied in the context of driving cessation to measure occupational performance related to loss of driving. During the interview, participants identify activities and roles that are difficult because of driving cessation, including daily living activities, community mobility, and social participation. Participants then select up to five occupational performance problems and rate performance for each item on a 1–10 scale, with higher scores indicating better performance. The primary endpoint is the post-intervention COPM performance score. The primary analysis will compare post-intervention COPM performance scores between groups using ANCOVA, adjusting for the baseline COPM performance score.

4.5 Secondary outcomes

1) Readiness for driving cessation

Readiness will be assessed using the Japanese version of the Assessment of Readiness for Mobility Transition (ARMT-J). The ARMT is a questionnaire assessing emotional and attitudinal readiness when mobility changes are required because of aging. The ARMT-J consists of 11 items in three subscales: Anticipatory Anxiety (AA), Perceived Burden (PB), and Adverse Situations (AS). Each item is rated on a five-point Likert scale from 1 (“strongly disagree”) to 5 (“strongly agree”), and results are interpreted using the mean score for each subscale.

2) Quality of life: Medical Outcomes Study 12-Item Short-Form Health Survey, version 2 (SF-12)

The SF-12 is a reliable and valid measure of health-related quality of life. It yields eight subscales: physical functioning, role physical, bodily pain, general health, vitality, social functioning, role emotional, and mental health. These can be summarized as the Physical Component Summary (PCS), Mental Component Summary (MCS), and Role–Social Component Score (RCS). Higher scores indicate better health-related quality of life. PCS, MCS, and RCS scores will be analyzed in this study.

3) Depressive symptoms: 15-item Geriatric Depression Scale (GDS-15)

The GDS-15 is a reliable and valid screening measure for depression. It consists of 15 questions answered “yes” or “no,” making it easy to complete and score. Scores of 5 or higher indicate depressive tendency, and scores of 10 or higher indicate a depressive state.

4) Environmental factors: Comprehensive Environmental Questionnaire for the Elderly (CEQ)

The CEQ is a reliable and valid questionnaire intended to assess environments that support quality of life among community-dwelling older adults. It contains 14 items in three domains: safe living environment (six items), social interaction environment (six items), and family environment (two items). Items are rated on a four-point scale from 1 (“not at all”) to 4 (“sufficient”). Domain and total scores are calculated by summation; higher scores indicate greater satisfaction with the environment.

5) Life-Space Assessment (LSA; prospective survey only)

The LSA captures physical activity through the concept of life space. It evaluates whether, how frequently, and how independently a person moves within the bedroom, home, area immediately outside the home (such as a garden or apartment grounds), neighborhood, town, and beyond town. Scores range from 0 to 120. Administration takes approximately 10 minutes, and reliability and validity have been established.

6) Evaluation of powered mobility devices

Powered mobility devices will be practiced and evaluated on the mobility-device course at the study institution. If a device is ultimately to be used in the community, a specialist vendor will provide further support and evaluation and determine whether its use is appropriate.

4.6 Other general information

- Age
- Sex
- Diagnosis
- Number of days since onset
- Availability of a person who can provide mobility support
- Severity of motor paralysis (Fugl-Meyer Assessment; FMA)
- Cognitive function (Mini-Mental State Examination; MMSE)
- Independence in activities of daily living (Functional Independence Measure; FIM)

4.7 Safety assessment items

Blood pressure, pulse, and peripheral oxygen saturation will be measured before and after assessment.

4.8 Study discontinuation

**Discontinuation criteria:**

- The participant requests withdrawal or withdraws consent.
- The participant is found after enrollment not to meet the eligibility criteria.
- Continuation of assessment is judged inappropriate because the primary disease has worsened.
- Continuation of assessment becomes difficult because of worsening comorbidities.

**Dropout criterion:**

- The participant dies.

4.9 Statistical methods

All data will be entered into Excel and analyzed using R version 4.5.2 (R Foundation for Statistical Computing, Vienna, Austria). Continuous variables will be summarized as means and standard deviations, and categorical variables as frequencies and proportions. Normality of continuous variables will be assessed using the Shapiro–Wilk test.

The primary analysis will focus on COPM performance. Between-group differences in post-intervention COPM performance will be examined using analysis of covariance (ANCOVA), with the pre-intervention score as a covariate. Changes from pre-intervention to post-intervention and to 1, 3, and 6 months after discharge will be analyzed using a mixed-effects model with group, time, and the group-by-time interaction as fixed effects. Because allocation is conducted at the ward level and only two ward clusters are included, cluster effects will not be statistically adjusted; findings will be interpreted as exploratory, and the possible influence of background differences between wards will be considered.

Secondary outcomes will be analyzed mainly using descriptive statistics, and trends in change will be examined as supportive evidence. Between-group comparisons at each time point will be presented using appropriate descriptive statistics. Because this is a pilot study, formal hypothesis testing of secondary outcomes is not the primary objective. Associations between ARMT-J scores and baseline characteristics will be explored using Pearson correlation coefficients. The significance level for the primary analysis will be p < 0.05. If missing data occur, multiple imputation will be considered before analysis.

Standardized data collection forms will be used for questionnaire assessments and will be available by contacting the responsible author when necessary. Analyses will follow a modified intention-to-treat principle and include participants with outcome data at one or more time points, regardless of protocol adherence.

5. Policy for Selecting Participants

5.1 Target population

Participants will be patients with acquired brain injury who are hospitalized or receiving outpatient care at a convalescent rehabilitation center in Shizuoka Prefecture, Japan; have undergone an automobile driving assessment; and have not been able to resume driving. Participants will be aged 55 years or older. This age threshold was selected to correspond with the population used in the ARMT-J study.

5.2 Inclusion criteria

1. Patients who, after receiving an adequate explanation of the study and demonstrating sufficient understanding, provide written consent voluntarily.
2. Patients aged 55 years or older at the time of consent.
3. Individuals who have ceased driving under an ordinary motor vehicle driver’s license.

5.3 Exclusion criteria

1. Individuals with conditions designated by the Public Safety Commission as disqualifying conditions for driving resumption, including epilepsy, schizophrenia, or dementia.
2. Individuals with clear cognitive impairment and an MMSE score of 24 or lower.
3. Patients with aphasia who cannot adequately understand the assessments.

5.4 Rationale for the criteria

The criteria were established to ensure participant safety and define the study population.

5.5 Selection process

Selection will follow the allocation procedure described in Section 4.2.1.

6. Number of Participants, Types of Materials and Information, and Study Sites

6.1 Number of participants

Because this is a single-center pilot and exploratory study with allocation determined by ward admission, the sample size was determined primarily on the basis of recruitment feasibility at the study site. A formal power calculation for the planned ANCOVA was not performed because reliable prior estimates of the intervention-specific between-group effect and the correlation between baseline and post-intervention COPM performance scores were not available.

The post-intervention COPM performance score is the primary endpoint. The primary analysis will estimate the between-group difference in post-intervention COPM performance scores using ANCOVA, adjusting for the baseline COPM performance score. A target sample size of 30 participants with evaluable primary outcome data was planned. To account for incomplete assessments and loss to follow-up, up to 40 participants may be enrolled. Equal numbers in the two groups are not assumed because group assignment is determined by ward admission.

This sample size is not intended to provide definitive statistical power for establishing intervention effectiveness or for accounting statistically for ward-level clustering. The adjusted between-group difference and its 95% confidence interval will be reported. Estimates of effect magnitude, variability, recruitment, retention, and data completeness will be used to inform the design and formal sample size calculation of a future adequately powered trial.

6.2 Types of materials and information

Materials and information obtained during the study will be recorded on paper and subsequently entered into a computer maintained at the study institution.

6.3 Study sites

CLASP-AID and the related assessments will be conducted in the occupational therapy room of JA Kyosai Nakaizu Rehabilitation Center. When an alternative mobility device is needed as a substitute for automobile driving, assessment and intervention will be performed using the institution’s mobility course. Alternative mobility devices will be evaluated using wheelchair testing (WCT).

7. Handling of Personal Information

7.1 Anonymization

Anonymization will be performed.

7.2 Method of anonymization

Data will be anonymized using codes that can be linked to a separately maintained correspondence table (pseudonymization).

7.3 Information management and storage

- Collected data will be used solely for research. Confidentiality will be strictly maintained, and data will be securely stored under the supervision of the institution’s ethics committee in a locked cabinet in the writing room.
- When research findings are presented at academic conferences or published in journals, interview data will be statistically processed so that individuals cannot be identified.
- When data are exchanged with co-investigators outside the institution, identifying information will be encrypted to prevent disclosure of personal information.

7.4 Disclosure of personal information

If a participant requests disclosure of personal information, materials concerning the study plan and methods will be provided to the extent that doing so does not interfere with the protection of other participants’ personal information or the originality of the study. The participant information sheet will instruct participants to contact the study contact listed at its end.

8. Procedures and Methods for Informed Consent

8.1 Written informed consent

The principal investigator and co-investigators will provide participants with the information and consent documents approved by the ethics committee, explain the study sufficiently in writing and orally, and obtain voluntary written consent.

If information that may affect a participant’s consent becomes available, or if the implementation plan or another matter affecting consent is changed, the principal investigator and co-investigators will promptly provide the relevant information and confirm in advance whether the participant wishes to continue. The information and consent documents will be revised after prior ethics committee approval, and renewed consent will be obtained. The documents will contain all information required by applicable ethical guidelines.

8.2 Oral informed consent and its documentation

The principal investigator and co-investigators will provide participants with a sufficient oral explanation concerning study participation and confirm their voluntary oral agreement. The date of consent, method of explanation, person providing the explanation, and matters agreed to will be documented in the medical record.

If information that may affect consent becomes available, or if the implementation plan or another matter affecting consent is changed, participants will be informed promptly, their willingness to continue will be confirmed, and this process will be documented in the medical record.

Information included in the informed-consent process

- Overview of the study, including purpose, methods, and duration
- Voluntary nature of participation
- Assurance that refusal to participate will not result in disadvantage
- Name of the research institution and names of the head of the institution and principal investigator
- Availability of the study protocol and materials concerning study methods for acquisition or review
- Handling of personal information and procedures for its disclosure
- Scope of use of the collected data
- Potential benefits and disadvantages of participation
- Contribution of the study to the relevant field and to society
- Study discontinuation and refusal
- Contact point for consultations by participants and related persons

Potential participant burdens are described in Section 13.2, “Foreseeable risks.” If information or protocol changes that may affect consent arise, the principal investigator and co-investigators will promptly inform participants, confirm their willingness to continue, obtain prior ethics committee approval for revisions to consent-related documents, and obtain renewed consent. Consent documents will contain the information required by applicable ethical guidelines.

9. Procedures and Methods When Consent Is Obtained from a Legally Authorized Representative

Proxy consent means consent provided on behalf of the participant. Patients with clear cognitive decline or aphasia are not eligible for this study; therefore, proxy consent is not applicable.

10. Procedures for Obtaining Informed Assent

Not applicable because this study does not involve children.

11. Future Use of Specimens and Information Obtained from Participants

Information obtained from participants may be used in future research that is not specified at the time consent is obtained. If the present work develops into a larger study, information obtained in this study may be used in that future research. Participants will be informed of the general purpose of such use.

After completion of this study, if participants have consented to the use of specimens or information in future research, the materials will be stored under the responsibility of the institution’s ethics committee. Before any newly planned study is implemented, an application will be submitted to and reviewed by the relevant ethics committee or clinical trial review committee. Even after consent, if the participant or legally authorized representative withdraws consent, specimens and information associated with the study will be disposed of in accordance with that person’s wishes.

12. Storage and Disposal of Specimens and Information

Except when participants have consented to long-term storage and future use, specimens and information will be retained until the later of the following dates: at least five years after study discontinuation or completion, or three years after publication of the study results. Paper documents will be shredded. Electronic media such as USB drives will be disposed of by completely deleting the data and physically destroying the media.

13. Burdens, Risks, and Benefits for Participants

13.1 Foreseeable benefits

Although this is an intervention study, there is currently no established evidence demonstrating the intervention’s effectiveness; therefore, participation may not provide a direct benefit. The findings may contribute to future advances in medical care. Participants who cooperate with all three post-discharge questionnaire surveys will receive a QUO prepaid card valued at JPY 1,000.

13.2 Participant burden and foreseeable risks

Participants in the intervention group will receive an educational program supporting adaptation after driving cessation that was not previously provided in a structured manner. Participation may create a burden. However, follow-up after driving cessation has previously been provided at the discretion of individual therapists, and the addition of this structured program is therefore not considered to create an ethical problem. If fatigue or another problem occurs during participation, a break will be provided and discontinuation of the study will be considered.

The study may include consideration of powered wheelchairs or mobility scooters. Because falls are possible on the mobility course, a therapist will remain beside the participant and be ready to provide support at all times. To prevent accidents, practical assessments will be conducted only on the mobility course or on a driving course when no vehicles are operating. The therapist will manage speed reduction before steps and slopes to prevent falls. A helmet will be provided to participants who wish to wear one. Driving assessment will not be conducted while other vehicles are operating alongside the participant.

13.3 Response to an accident

If an accident occurs outdoors, staff will first contact the Center using the mobile telephone they carry and request additional staff assistance. Subsequent accident management will follow the incident-response procedures of the institution’s Safety Management Committee.

14. Response to Consultations from Participants and Related Persons

Consultations will be handled in accordance with the Nakaizu Rehabilitation Center consent form.

15. Provision of Medical Care After Completion of the Study

Not applicable.

16. Handling of Adverse Events

The Center maintains insurance coverage for accidents. If an accident occurs, it will be handled through the Center’s insurance coverage. If an accident occurs outdoors, staff will first contact the Center using the mobile telephone they carry and request additional staff assistance. Subsequent management will follow the incident-response procedures of the institution’s Safety Management Committee.

17. Compensation for Health Injury Caused by the Study

Not applicable.

18. Handling of Research Results Pertaining to Participants

Not applicable.

19. Content and Method of Reports to the Head of the Research Institution

If the principal investigator intends to conduct the study under a modified protocol, the protocol will be amended in advance and reported to the head of the research institution through the ethics committee secretariat. Other matters requiring reporting to the head of the institution will also, in principle, be submitted through the ethics committee secretariat.

20. Outsourcing of Study-Related Activities

No study-related activities will be outsourced.

21. Conflicts of Interest Related to the Study

There are no conflicts of interest. The study is funded by a 2023 research grant from the Toyota Foundation.

22. Monitoring and Auditing

Not applicable.

23. References

1. Takehara I, et al. Survey of the resumption of automobile driving among patients with stroke. *Journal of the Japanese Council of Traffic Science*. 2009;9(1):51–55. [In Japanese]
2. Nasu S, et al. Differences in occupational participation between groups who resumed and ceased driving after stroke in a mountainous region: A questionnaire survey. *Journal of Occupational Behavior*. 2022;26(3):139–146. [In Japanese]
3. Nasu S, et al. Effects of differences in decision-making factors for driving cessation after acquired brain injury on occupational participation: A preliminary questionnaire study. *Japanese Occupational Therapy Research*. In press. [In Japanese]
4. Nasu S, et al. Experiences of individuals and families after automobile driving cessation following acquired brain injury: A qualitative meta-study. *Japanese Occupational Therapy Research*. 41(6):663–675. [In Japanese]
5. Stowe J. *Mobile aging: A randomized trial of mobility transition counseling for high-risk older adults* [doctoral dissertation]. University of Missouri–Columbia; 2014.
