# Supplementary material for: Study protocol for a non-randomized trial of community participation support for people with Acquired Brain Injury who have ceased driving in Japan
Source: PLoS One. 2026 Nov 10;21(11):e0362487. doi: 10.1371/journal.pone.0362487 (PMC13750569; doi:10.1371/journal.pone.0362487)
Supplement: Supplementary file 4 [file pone.0362487.s004.docx]

**同 意 説 明 文 書（利用者・患者様用）**

研究課題：後天性脳損傷後の自動車運転中断者に対しての地域社会参加支援の効果　- 日本における非ランダム化研究 -

研究者名：那須　識徳

所　　属：農協共済中伊豆リハビリテーションセンター

　　　　　東京都立大学　人間健康科学研究科　作業療法科学域　客員研究員

この同意説明文書は、研究課題【後天性脳損傷後の自動車運転中断者に対しての地域社会参加支援の効果】について、その趣旨を十分にご理解いただくことを目的として作成されたものです。この研究に参加・協力していただけるかどうかは、あなたの自由意志によって決めていただきます。

1. **研究の目的**

脳卒中後や加齢に伴う自動車運転の中断は、社会的孤立やうつ病といった２次的な健康被害の要因となることが知られています。海外での高齢者対象の研究では、運転など個人の移動手段を変更するためには、将来の移動手段について計画しておく必要があり、予め移動手段の変更について計画ができていると、うつ病などの健康面の問題は起こりにくくなると言われています。さらに、運転を辞めたとしても、地域で自分の行いたい活動を継続できている場合、運転の再開率は高まることも知られています。

本研究は自動車運転が一時的にでも中断する可能性がある方を対象に**運転中断後の地域生活支援プログラム：C**ommunity **L**ife **S**upport **P**rogram for Individuals with **A**cquired Brain **I**njury after **D**riving Cessation **(CLASP-AID)**と通常の運転中断支援との比較を行うことで、運転中断後の支援の有用性について検証することを目的としています。

2. **研究の方法（研究期間含む）**

本研究では、自動車運転が一時的にでも中断する可能性がある方を対象に、まず支援開始時に必要な評価を受けて頂きます。その後、運転中断支援を受けて頂きます（１回30分～40分程度）。退院時には、ご家族や近親者にも運転中止後の生活についての注意事項の説明を聞いて頂きます。（１回30分程度）。具体的には、自動車運転を中止した後の生活について、訓練士と考える場を設けて具体的な退院後の生活について検討することとなります。必要な場合には、公共交通や地域移動サービスの紹介、セニアカーなどの代替移動手段の紹介を行います。すべての支援が終わったら、再度評価を行い、退院後１か月、３ヵ月、６ヵ月に、退院後の生活がどうであったか、アンケートを受けて頂くこととなります。本研究は、支援の内容がA群とB群に分かれております。研究を実施する都合上、A群とB群の支援内容の詳細を述べることはできませんが、A群もB群も運転中断後の生活について検討する予定です。研究参加による不利益を最大限に減らすために、研究終了後にもう一方の群で実施した内容を知ることは可能ですし、必要であれば実施した内容の資料を渡すことも可能です。必要な場合は担当訓練士か、研究責任者までお問合せください。

なお、セニアカーの利用にあたっては、退院後福祉用具業者が使用に関しての評価を行うことが一般的です。当センターでは、あくまでも使い方や乗り心地の確認と体験程度になることをご了承ください。

**研究期間**：2024年９月1日～202７年３月31日

**【患者様自身に行っていただく評価】**：自己記入式（患者様に行って頂く評価）

- 運転中断の準備性の評価：Assessment of Readiness for Mobility Transition ( ARMT-J )
- 生活の質の評価：SF-12
- 生活空間の評価：Life space assessment
- うつ病の評価：Geriatric Depression Scale(GDS-15-J)
- 包括的環境要因調査表：Comprehensive Environmental Questionnaire for the Elderly(CEQ)

※ 運転中断時にセニアカーなどの電動モビリティー機器が必要な場合は

　 練習はモビリティー機器のコースを利用して練習を行いますが、最終

的に地域で使用する場合は、専門業者の支援と評価を受けて頂きます

**【一般情報の収集】**　※ こちらはスタッフが記載致します。

- 性別、年齢、疾患名、発症からの日数、移動支援者の有無、教育歴、運転歴、FIM（日常生活の自立度）、FMA（運動麻痺の程度）、MMSE（認知機能の程度）

1. **研究倫理**

本研究は、中伊豆リハビリテーションセンターの倫理審査（社中総発第55号）の承認を得て実施しています。

2. **研究への参加と取り止めの自由について**

本研究では、人権の基本である自由意思を第一優先事項とし、本研究への参加はあくまで**個人の自由意思**によるものとします。いつでも参加を取り止めることが可能であり、もし途中で**参加を取り止めたとしても不利益を被ることはありません**。研究対象者から参加への中止の申し出があった場合などは、本研究のために収集した情報はすべて破棄することとします。なお、研究同意の撤回がある場合は、研究責任者に直接申し付けていただくか、担当訓練士まで申しつけください。後日研究同意撤回書に署名を頂き、研究の撤回とさせて頂きます。

3. **個人情報保護**

研究によって得られた個人情報は本研究のみで使用し、学会や論文などにおいて結果を公表する際に個人が同定されないように扱います。また、紙面のデータは鍵付き書庫に保管されたのちに、破棄いたします。USBなどの電子媒体は研究終了後に保管期間を過ぎたらデータを消去し、さらに電子媒体を破壊いたします。保管期間は結果公表後３年または、研究終了後から５年といたします。

4. **得られたデータの利用範囲、研究成果の発表方法**

　本研究は学術論文および学会発表や論文作成に使用する予定です。 データ収集後の分析から発表時に至るまで、個人が特定されないように匿名化された状態でデータは扱います。データの分析上外部の者とデータを共有することがありますが、個人が同定できないようにデータの処理を行います。

　また，得られたデータに関しては、将来的に、より大規模な研究に発展した場合に改めて利用する可能性がございます。その場合には、改めて説明を行った上で同意が得られた場合のみ、データの使用を行います。

5. **研究に参加することで得られる利益と不利益**

　今回我々が行う２つの支援方法は、先行研究などを参考に計画しましたが、その効果を実証した研究はこれまでになく、そのような点から言えば、科学的な根拠に乏しいものとなっております。そのため、本研究によって得られる利益はありません。ただし、本研究を通じて、これらの介入になんらかの効果が得られれば、運転を中止したことで生じる問題を１つでも減らすことに貢献できるのではないかと考えております。尚、アンケート調査にも同意された場合は、アンケートの送付をもってQuoカード1000円分を送付させて頂きます。

不利益としては、介入効果を確認するために１つ１つの評価を丁寧に行うという観点から、通常のリハビリテーションよりも少し時間がかかり疲労を伴う可能性があるという点が挙げられます。疲労が生じた場合は必要に応じて適宜休憩時間を設けます。また、シニアカーなどの代替移動機器が必要な方に対しては実機による体験も行います。シニアカーは道路交通法上歩行者同様の扱いなので、安全性は自転車よりは高いと考えますが、転倒リスクはゼロではありません。転倒に備え訓練士は常にセニアカーの横で待機し、必要に応じて速度のコントロールを行うと同時に、**希望者には**セニアカー実施時にヘルメットを着用します。また、転倒時には当センターで加入している保険にて対応します。屋外において事故が起きた時の具体的な対応としては、まず持参している携帯電話にてセンターに電話を行い、応援職員を呼んで対応します。その後の事故処理は当院の安全管理委員会のインシデント対応に準ずるものとします。

6. **研究の当該領域・社会に対する貢献**

　本研究で、運転を一時的にでも中止する必要がある方への支援体制を確立することで、今後の運転リハビリテーションにおいて運転中断者の支援の一助になると考えます。

7. **研究に関わる経費：**通常の診療等の料金はかかりますが、本研究の参加に関して特に過剰に経費がかかることはございません。
8. **問い合わせ・連絡先**

**研究責任者氏名**：那須　識徳

**所　属：**中伊豆リハビリテーションセンター

**所在地：**［非公開］

**連絡先：**TEL ［非公開］

メールアドレス：sa-nasu@janrc.or.jp

**研　究　協　力　同　意　書**

所属機関名　農協共済中伊豆リハビリテーションセンター　作業療法科

　　　　　　東京都立大学　人間健康科学研究科　作業療法科学域

**研究代表者名　　那　須　識　徳**

問い合わせ先　TEL ［非公開］

　このたび、私は、「後天性脳損傷後の自動車運転中断者に対しての地域社会参加支援の効果　- 日本における非ランダム化研究 -」に参加するにあたり、

令和　　年　　月　　日に　　　　　　　　　　　　　　　より以下の

項目について文書および口頭にて説明を受けました。

- 研究の目的および方法
- 研究期間
- 研究への参加は任意であること
- 研究に参加することに同意しない場合でも、不利益を受けないこと
- 研究に参加することに同意した場合でも、随時これを撤回でき、不利益を受けないこと
- 個人情報保護（匿名化の方法）
- 得られたデータの利用範囲
- 研究成果の発表
- 研究に参加することで得られる利益と不利益
- 研究の当該領域・社会に対する貢献
- 研究に関わる経費
- 問い合わせ・連絡先

　その結果、上記の内容を十分理解しましたので、研究に参加することに同意します。

（令和　　年　　月　　日）

氏　名

同　意　撤　回　文　書

農協共済中伊豆リハビリテーションセンター

リハビリテーション部　作業療法科　　　　那須　識徳　殿

研究課題名：「後天性脳損傷後の自動車運転中断者に対しての地域社会参加支援

の効果　- 日本における非ランダム化研究」

　私は、上記研究への参加に同意しましたが、同意を撤回します。

　　　年　　　月　　　日

患者様　氏名：	　　　　　　　　　　　　　（自署）

【確認者の署名欄】

　　　年　　　月　　　日

確認者 氏名：　　　　　　　　　　　　（自署）
