# Supplementary material for: Study protocol for a non-randomized trial of community participation support for people with Acquired Brain Injury who have ceased driving in Japan
Source: PLoS One. 2026 Nov 10;21(11):e0362487. doi: 10.1371/journal.pone.0362487 (PMC13750569; doi:10.1371/journal.pone.0362487)
Supplement: Supplementary file 5 [file pone.0362487.s005.docx]

Participant Information Sheet

For service users and patients

English translation

Study title: Effects of Community Participation Support for People Who Have Ceased Driving after Acquired Brain Injury — A Non-Randomized Study in Japan

Researcher: Satonori Nasu

Affiliations: JA Kyosai Nakaizu Rehabilitation Center; Visiting Researcher, Department of Occupational Therapy, Graduate School of Human Health Sciences, Tokyo Metropolitan University

This information sheet is intended to help you fully understand the purpose of the study, “Effects of Community Participation Support for People Who Have Ceased Driving after Acquired Brain Injury.” You are free to decide whether to participate in and cooperate with this study.

# 1 Purpose of the study

Stopping driving after a stroke or as a result of aging is known to contribute to secondary health problems, such as social isolation and depression. Studies of older adults outside Japan have indicated that people need to plan their future mobility when changing how they get around, including how they travel by car. Planning these changes in advance is reported to make health problems such as depression less likely. It is also known that people who continue the activities they wish to do in their community after stopping driving have a higher rate of resuming driving.

This study aims to examine the usefulness of support after driving cessation by comparing the Community Life Support Program for Individuals with Acquired Brain Injury after Driving Cessation (CLASP-AID) with usual support for driving cessation. The study is intended for people who may need to stop driving, even temporarily.

# 2 Study methods and study period

If you may need to stop driving, even temporarily, you will first undergo the necessary assessments at the start of support. You will then receive support for driving cessation, with each session lasting approximately 30–40 minutes. At discharge, your family members or close relatives will also be asked to attend an explanation of points to consider in daily life after driving cessation, lasting approximately 30 minutes.

You will have opportunities to discuss life after stopping driving with a therapist and consider specific plans for life after discharge. If necessary, information will be provided about public transportation, local mobility services, and alternative means of mobility, such as mobility scooters.

# Study methods continued

After all support has been completed, you will be assessed again. At 1, 3, and 6 months after discharge, you will be asked to complete questionnaires about how life has been after discharge.

The support provided in this study differs between Group A and Group B. For the purposes of conducting the study, we cannot explain the detailed support provided to each group. However, both groups will consider life after driving cessation. To minimize disadvantages associated with participation, you may learn about the support provided to the other group after the study has ended. If needed, you may also receive materials describing that support. Please contact your treating therapist or the principal investigator if you would like this information or these materials.

When a mobility scooter is to be used after discharge, an assistive device supplier will generally assess its use. Please understand that activities at our center are limited to learning how to use the device, checking how comfortable it feels, and trying it out.

Study period: September 1, 2024 to March 31, 2027

## Assessments you will complete yourself

- Readiness for driving cessation: Assessment of Readiness for Mobility Transition (ARMT-J)
- Quality of life: SF-12
- Life-space mobility: Life Space Assessment
- Depression: Geriatric Depression Scale (GDS-15-J)
- Environmental factors: Comprehensive Environmental Questionnaire for the Elderly (CEQ)

If a powered mobility device, such as a mobility scooter, is needed after driving cessation, you will practice using it on the mobility device course. When you eventually use the device in the community, you will receive support and an assessment from a specialist provider.

## General information collected by staff

Sex, age, diagnosis, time since onset, availability of a person to assist with mobility, educational history, driving history, FIM (independence in daily activities), FMA (degree of motor paralysis), and MMSE (cognitive function).

# 3 Research ethics

This study is conducted with approval from the ethics review committee of Nakaizu Rehabilitation Center (approval reference: Sha-Chu-So-Hatsu No. 55).

# 4 Voluntary participation and withdrawal

Respect for free choice, a basic human right, is our first priority. Participation in this study is entirely voluntary. You may stop participating at any time and will not be disadvantaged if you do so. If you request to stop participating, all information collected for this study will be destroyed. To withdraw your consent, please contact the principal investigator directly or inform your treating therapist. You will subsequently be asked to sign a consent withdrawal form to withdraw from the study.

# 5 Protection of personal information

Personal information obtained through this research will be used only for this study. When findings are presented at conferences or published in journal articles, the information will be handled so that individuals cannot be identified. Paper records will be kept in a locked cabinet and subsequently destroyed. After the study has ended and the storage period has elapsed, data on electronic media such as USB drives will be deleted, and the media will also be physically destroyed. The storage period is three years after publication of the results or five years after completion of the study.

# 6 Use of data and dissemination of findings

The findings of this study will be used for academic publications, conference presentations, and manuscript preparation. From analysis after data collection through to presentation or publication, data will be handled in an anonymized form so that individuals cannot be identified. Data may be shared with people outside the research team for analysis; in such cases, the data will be processed so that individuals cannot be identified.

The data obtained may also be used again if this work develops into a larger study in the future. In that case, the research will be explained to you again, and your data will be used only if you give your consent.

# 7 Benefits and disadvantages of participation

The two types of support in this study were developed with reference to previous research. However, their effectiveness has not yet been demonstrated in a study, and the scientific evidence supporting them is therefore limited. For this reason, participation in this study will not provide a benefit to you. Nevertheless, if the study identifies an effect of these interventions, it may contribute to reducing problems caused by stopping driving. If you also agree to the questionnaire survey, a QUO gift card worth 1,000 yen will be sent to you when you return the questionnaire.

# Benefits and disadvantages continued

One disadvantage is that the assessments may take slightly longer than usual rehabilitation and may cause fatigue, because each assessment is conducted carefully to examine the effects of the intervention. Rest breaks will be provided as needed if you become tired.

If you need an alternative mobility device such as a mobility scooter, you will also have an opportunity to try the actual device. Under the Road Traffic Act, mobility scooters are treated in the same way as pedestrians, and we therefore consider them safer than bicycles. However, the risk of falling is not zero. To prepare for this risk, a therapist will remain beside the mobility scooter at all times and control its speed when necessary. Participants who wish to wear a helmet will do so while using the scooter. Falls will be handled under the insurance held by our center.

If an accident occurs outdoors, staff will first use the mobile phone they are carrying to contact the center and request assistance from additional staff. Subsequent handling of the accident will follow the incident response procedures of the center’s Safety Management Committee.

# 8 Contribution to the field and to society

By establishing a support system for people who need to stop driving, even temporarily, this study may help support people who cease driving within future driving rehabilitation services.

# 9 Costs associated with the study

You will be responsible for the usual costs of medical care and related services, but participation in this study will not involve any substantial additional costs.

# 10 Questions and contact information

Principal investigator: Satonori Nasu

Affiliation: Nakaizu Rehabilitation Center

Address: [redacted]

Telephone: [redacted]

Email: sa-nasu@janrc.or.jp

Consent to Participate in Research

English translation

Institutions: Department of Occupational Therapy, JA Kyosai Nakaizu Rehabilitation Center; Department of Occupational Therapy, Graduate School of Human Health Sciences, Tokyo Metropolitan University

Principal investigator: Satonori Nasu

Contact telephone: [redacted]

In considering participation in the study entitled “Effects of Community Participation Support for People Who Have Ceased Driving after Acquired Brain Injury — A Non-Randomized Study in Japan,” I received written and oral explanations of the following items from the person named below on the date indicated.

Date of explanation: Reiwa ______ (year) / ______ (month) / ______ (day)

Person providing the explanation: ___________________________________

☐ Purpose and methods of the study

☐ Study period

☐ Participation in the study is voluntary

☐ Declining to participate will not result in any disadvantage

☐ Consent may be withdrawn at any time without disadvantage, even after agreeing to participate

☐ Protection of personal information and methods of anonymization

☐ Scope of use of the data obtained

☐ Dissemination of study findings

☐ Benefits and disadvantages of participation

☐ Contribution of the study to the field and to society

☐ Costs associated with the study

☐ Questions and contact information

I have fully understood the information described above and agree to participate in this study.

Date: Reiwa ______ (year) / ______ (month) / ______ (day)

Name: __________________________________________________________

Withdrawal of Consent

English translation

To: Satonori Nasu
Department of Occupational Therapy, Rehabilitation Department
JA Kyosai Nakaizu Rehabilitation Center

Study title: Effects of Community Participation Support for People Who Have Ceased Driving after Acquired Brain Injury — A Non-Randomized Study in Japan

I previously agreed to participate in the study named above, but I now withdraw my consent.

Date: ______ (year) / ______ (month) / ______ (day)

Participant’s name, handwritten: ___________________________________

# Confirmation of withdrawal

Date: ______ (year) / ______ (month) / ______ (day)

Name of the person confirming withdrawal, handwritten:

________________________________________________________________
